# AAV-mediated gene therapy in a model of SLC13A5 citrate transporter disorder rescues epileptic and metabolic phenotypes

**DOI:** 10.1172/JCI197503

**Published:** 2026-02-19

**Authors:** Lauren E. Bailey, Raegan M. Adams, Morgan K. Schackmuth, Irvin T. Garza, Krishanna Knight, Sydni K. Holmes, Meghan M. Eller, MinJae Lee, Rachel M. Bailey

**Affiliations:** 1 Center for Alzheimer’s and Neurodegenerative Diseases, University of Texas Southwestern Medical Center, Dallas, Texas, USA.; 2 Graduate School of Biomedical Sciences, University of Texas Southwestern Medical School, Dallas, Texas, USA.; 3 Department of Health Data Science and Biostatistics, Peter O’Donnell Jr. School of Public Health, and; 4 Department of Pediatrics, University of Texas Southwestern Medical Center, Dallas, Texas, USA.

**Keywords:** Genetics, Neuroscience, Epilepsy, Gene therapy, Monogenic diseases

## Abstract

SLC13A5 citrate transporter disorder is a rare epileptic encephalopathy caused by loss-of-function pathogenic variants in the *SLC13A5* gene. Loss of sodium/citrate cotransporter (NaCT) function causes a severe early-life epilepsy resulting in lifelong developmental disabilities and increased extracellular citrate. Current antiseizure medications may reduce seizure frequency, yet more targeted treatments are needed to address the epileptic and neurodevelopmental SLC13A5 phenotype. We performed preclinical studies in SLC13A5-deficient (KO) mice evaluating phenotype rescue with adeno-associated virus (AAV) vector carrying a functional copy of the human *SLC13A5* gene (AAV9/SLC13A5). Cerebrospinal fluid delivery of AAV9/SLC13A5 decreased extracellular citrate levels, normalized electrophysiologic and sleep architecture abnormalities, and restored resistance to chemically induced seizures and death. Treatment benefits were achieved with administration during early brain development and in young adult mice, indicating therapeutic efficacy across developmental and postdevelopmental stages. Comparison of delivery routes in young adult KO mice showed that higher brain targeting achieved with intra–cisterna magna delivery resulted in greater treatment benefit as compared with intrathecal lumbar puncture delivery. Together, these results support further development of AAV9/SLC13A5 for treating SLC13A5 citrate transporter disorder.

## Abstract 2

Bailey et al. report on preclinical studies supporting efficacy and tolerability of gene replacement therapy in a mouse model of SLC13A5 citrate transporter disorder.

## Graphical abstract

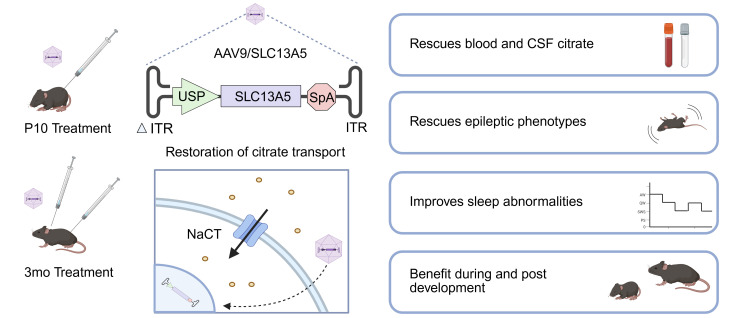

## Introduction

SLC13A5 citrate transporter disorder (also known as SLC13A5 epilepsy, SLC13A5 deficiency, or developmental epileptic encephalopathy-25 [DEE25]) is a rare autosomal recessive epileptic encephalopathy with seizure onset in the first day of life along with persistent intellectual and motor disabilities (1). Patients have multifocal epilepsy and cognitive and sleep abnormalities (2). The disease is caused by biallelic loss-of-function mutations in the *SLC13A5* gene (3). There are currently only symptomatic treatments, and a curative therapy for SLC13A5 deficiency is needed. Seizures are often pharmacoresistant and children have succumbed to seizure complications (4). The degree of neurodevelopmental disability and seizure burden vary, but most patients are severely affected, requiring 24/7 lifelong care (5). A therapy that addresses the underlying cause of the disorder would have a profound impact on the patients and their families’ well-being and decrease health care costs.

The *SLC13A5* gene codes for a sodium-dependent citrate transporter (NaCT) that has the highest expression in the liver, followed by brain, testis, kidney, and bones. In brain, NaCT is found in the cell membrane of neurons, astrocytes, and other cell types. Citrate plays a critical role in cellular energy metabolism, neurotransmitter synthesis, and lipid metabolism. To date, all tested mutations result in no or a greatly reduced amount of citrate transport (6). Patients with SLC13A5 deficiency show distinctive elevations of citrate in the plasma and cerebrospinal fluid (CSF) (7–9). Diminished intracellular citrate and/or elevated extracellular citrate may be the underlying cause of the frequent seizures and developmental disabilities in patients.

The *Slc13a5-*knockout (*Slc13a5*-KO) mouse lacking NaCT shows increased neuronal excitability and propensity for epileptic seizures, abnormal citrate levels in CSF and brain tissue, and impaired bone and tooth development (8, 10–12). Recently, we reported that young adult *Slc13a5-*KO mice have sleep deficits, similar to the sleep deficits observed in patients, which coincided with abnormal EEG power spectrum (2). While *Slc13a5-*KO mice model salient aspects of SLC13A5 citrate transporter disorder that can be utilized for therapeutic development, they lack overt behavioral, motor, or cognitive impairments that are seen in patients (6).

Gene therapy is a precision medicine where functional genetic material is delivered to cells that has the potential to address the underlying cause of a disorder rather than managing symptoms. Adeno-associated virus (AAV) vectors have efficient gene transfer, broad serotype-dependent tropism, low risk of insertional mutagenesis, and long-term transgene expression in transduced cells following a single injection (13). AAV-based gene therapies show great promise in treating neurological disorders, and currently 8 FDA-approved AAV gene therapies use different serotypes (14–17). AAV serotype 9 (AAV9) is most frequently used for CNS brain delivery because of its ability to achieve broad transgene transduction across the CNS of rodents and large animals, and injection into CSF transduces peripheral tissues in addition to the CNS (18, 19). Importantly, CSF AAV9 gene therapy is being utilized in active clinical trials for pediatric neurological disorders, such as giant axonal neuropathy, Rett syndrome, and spastic paraplegia 50 (20–22).While AAV vectors are small, with a packaging size less than 5 kilobases, the coding sequence of the *SLC13A5* gene is within this limit, making gene therapy an amenable approach (23).

Here we report the development of a self-complementary AAV9 vector delivering optimized *SLC13A5* (AAV9/SLC13A5) to treat SLC13A5 citrate transporter disorder, the design of which can be readily translated for clinical use. Preclinical efficacy studies were performed using CSF delivery in both pups and young adult *Slc13a5-*KO mice. Following intrathecal lumbar puncture (IT) administration of AAV9/SLC13A5 in *Slc13a5-*KO pups, blood and CSF citrate levels were decreased, brain activity and sleep abnormalities were normalized, epileptic discharges were decreased, and seizure onset sensitivity and severity were attenuated. In young adult mice, IT and intra–cisterna magna (ICM) administrations of AAV9/SLC13A5 were directly compared, and similar to adolescent mice, AAV9/SLC13A5 treatment decreased blood citrate levels, normalized EEG activity, improved sleep architecture, increased resistance to seizures, and protected against seizure-induced deaths. A greater treatment benefit in adult KO mice was achieved with ICM delivery, which also resulted in greater AAV9/SLC13A5 gene transfer in the adult brain. Importantly, AAV9/SLC13A5 administration in both pups and adult mice was safe and well tolerated.

## Results

### Design and functional testing of a gene vector expressing NaCT.

**Figure 1:**
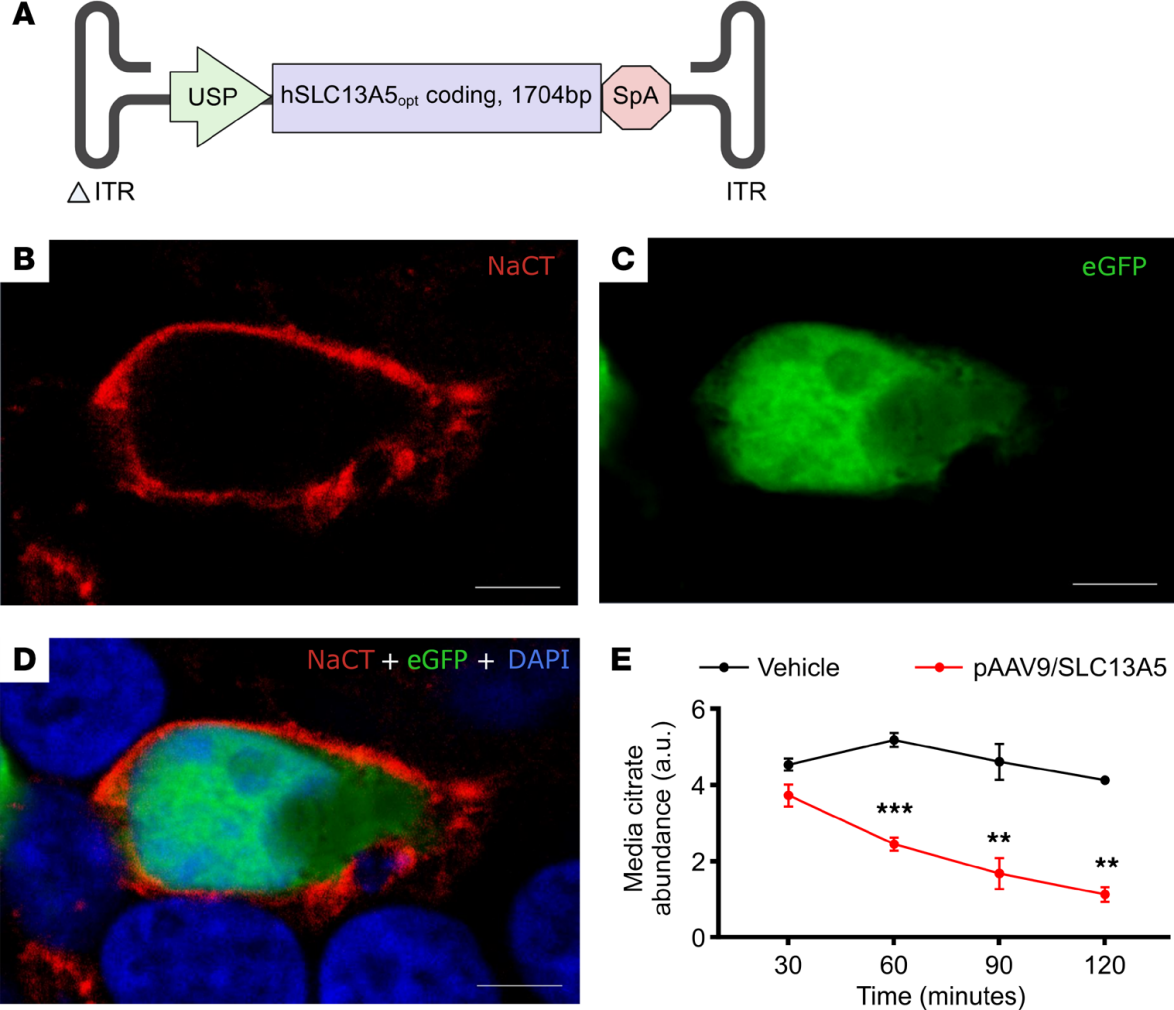
AAV/SLC13A5 vector design, expression, and function in cells. (**A**) Schematic of AAV9/SLC13A5 transgene. ITR, inverted terminal repeat. (**B**–**D**) Representative image of a HEK293T cell cotransfected with a plasmid expressing eGFP and pAAV/SLC13A5 showed that 48 hours after transfection NaCT staining was in a punctate pattern around the outer edge of the cell and that eGFP fluorescence, which was detected homogenously in the cytosol, did not colocalize with NaCT (**B**, NaCT; **C**, eGFP; **D**, merged NaCT, eGFP, and DAPI). Scale bar: 5 μm. (**E**) Uptake of citrate (1 μM) into HEK293T cells transfected with vehicle or pAAV9/SLC13A5. Citrate abundance in the media was assessed at 30, 60, 90, and 120 minutes after addition to wells via GC-MS. Two-way ANOVA, Šídák’s post hoc analysis; **P < 0.01, ***P < 0.001. *n* = 3 per treatment. Data shown as mean ± SEM. a.u., arbitrary units.

For downstream human application, we developed the AAV/UsP-SLC13A5 vector consisting of a ubiquitously expressed minimal synthetic JeT plus intron promoter (UsP) (24), driving the expression of codon-optimized human *SLC13A5* cDNA that has a synthetic polyA tail (Figure 1A) (25). Given AAV packaging constraints (26, 27), use of the short UsP allows self-complementary packaging of the 1.7 kb SLC13A5 coding sequence, which is predicted to stably transduce at least 10-fold more cells than single-stranded AAV (26, 27). The gene insert is bounded by the AAV2 ITRs, where 1 terminal repeat has the WT 144 nt sequence and the other ITR (ΔITR) is mutated to delete the AAV DNA resolution site and D sequence to direct preferential replication and packing of self-complementary AAV (scAAV) DNA sequences (28). We chose a scAAV design to promote faster and more stable onset of transgene expression and accommodate a smaller gene load without the need for exogenous stuffer sequences. While rapid expression is not strictly required for disease maintenance, earlier functional rescue may mitigate downstream network and developmental abnormalities in SLC13A5 deficiency. This construct was packaged into an AAV9 capsid, a clinically validated serotype with strong translational precedent and broad CNS tropism. Since SLC13A5 is expressed in both the brain (neurons and glia) and liver, use of a ubiquitous promoter would allow for expression in critical cell and tissue types.

Expression of the AAV/SLC13A5 vector plasmid was assessed using immunocytochemistry, as commercially available NaCT antibodies did not reliably identify a band via Western blot analysis. HEK293T cells lack NaCT expression and have naturally low transport of extracellular citrate, thus providing an applicable assay to test the functional potential of the SLC13A5 gene therapy vector in human cells. To assess localization, HEK293T cells were transfected with our pAAV/SLC13A5 plasmid and a plasmid expressing eGFP protein. eGFP is a soluble protein that is diffusely located in the cytoplasm and should not overlap with plasma membrane proteins. After 48 hours, cells were fixed, and immunocytochemistry was performed with antibodies that recognize human NaCT and eGFP, respectively, to verify that NaCT localized to the plasma membrane (Figure 1, B–D). To confirm vector-expressed NaCT transports citrate into cells, we transfected HEK293T cells with pAAV9/SLC13A5 or vehicle for 48 hours. We added 1 μM of citric acid to the cell culture media, and then a time course of collected media samples was assessed for citrate using gas chromatography-MS (GC-MS). Over 2 hours, cells transfected with vehicle showed very low baseline citrate transport, while pAAV9/SLC13A5 treatment decreased extracellular citrate levels by about 70% (Figure 1E), indicating increased citrate transport from the extracellular fluid into cells. Together these results demonstrate that pAAV9/SLC13A5 results in functional NaCT protein in human cells.

### AAV9/SLC13A5 treatment restores functional NaCT in Slc13a5-KO mice.

**Figure 2:**
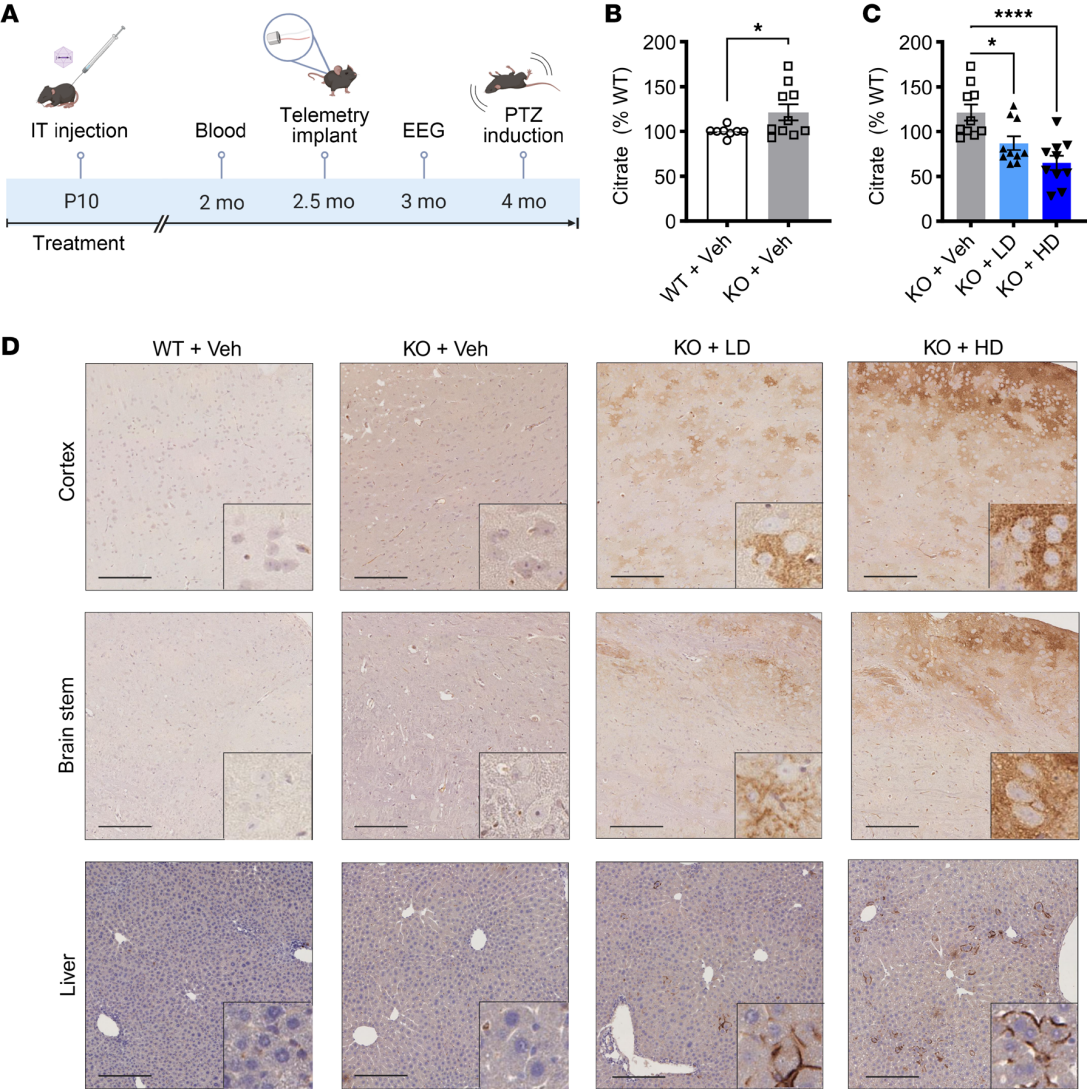
P10 IT administrations of AAV9/SLC13A5 gene therapy in *Slc13a5*-KO mice result in robust NaCT in the brain and decreased blood citrate levels. (**A**) Schematic of the efficacy study in *Slc13a5*-KO mice IT-injected at P10 with vehicle or 2 × 10^11^ vg (low dose; LD) or 8 × 10^11^ vg (high dose; HD) of AAV9/SLC13A5. (**B** and **C**) GC-MS detection of citrate levels 2 months posttreatment relative to WT controls. (**B**) Vehicle-treated WT and *Slc13a5*-KO mice. Student’s unpaired *t* test, *P < 0.05. (**C**) Vehicle-, AAV9/SLC13A5 LD–, and HD-treated KO mice. One-way ANOVA with Dunnett’s multiple comparisons test, *P < 0.05, ****P < 0.0001. *n* = 8 WT+Veh, 10 KO+Veh, 10 KO+LD, 10 KO+HD. Data shown as mean ± SEM. (**D**) Representative images of mouse brain and liver collected 4 months posttreatment and stained with SLC13A5/NaCT antibody in the cortex (top), brain stem (middle), and liver (bottom). Scale bar: 200 μm.

To test the hypothesis that broad CNS and peripheral delivery of the human SLC13A5 gene using AAV9 can provide an effective treatment for SLC13A5 citrate transporter disorder, we utilized homozygous *Slc13a5-*KO mice (10). Preclinical efficacy studies were performed in postnatal day 10 (P10) pups, comparing IT delivery of either 2 × 10^11^ vector genome (vg; low dose) or 8 × 10^11^ vg (high dose), and mice were followed up to approximately 4 months (mo) postinjection (Figure 2A). Vehicle-treated WT and KO littermates served as controls. We first determined if AAV9/SLC13A5 treatment resulted in functional NaCT by measuring blood citrate levels. This is clinically relevant, as patients have significantly elevated citrate levels in the blood and CSF. Similarly, *Slc13a5-*KO mice had significantly elevated citrate levels (Figure 2B). Treatment with AAV9/SLC13A5 significantly decreased plasma citrate levels in KO mice in a dose-dependent manner as compared with control KO mice, where at 2 mo postinjection low dose–treated mouse citrate levels were decreased to 87% ± 7.7% of WT levels and high dose–treated mice were decreased to 65% ± 8.0% (Figure 2C), supporting that NaCT function was restored.

We directly assessed NaCT protein expression using immunohistochemical analysis. In WT mice, staining was indistinguishable from that observed in vehicle-treated KO mice, indicating that the NaCT antibody does not recognize endogenous mouse NaCT protein and that the signal represents nonspecific background signal (Figure 2D). In contrast, P10 AAV9/SLC13A5-treated mice showed dose-dependent, widespread expression of NaCT throughout the brain and liver (Figure 2D and Supplemental Figure 1; supplemental material available online with this article; https://doi.org/10.1172/JCI197503DS1). Notably, the staining pattern was consistent with membrane localization as shown in the Figure 2D insets, supporting appropriate plasma membrane trafficking of the transgene-encoded NaCT protein.

### AAV9/SLC13A5 treatment is well tolerated in Slc13a5-KO mice.

Vector treatment in KO mice was well tolerated, with no adverse effects observed across treated animals. This was supported by normal body weight gain and maintenance, survival, and general activity in all groups (Supplemental Figure 2). We also assessed urinary citrate levels. Vehicle-treated WT and KO mice exhibited comparable urinary citrate levels, albeit with high variability within each group, suggesting no baseline difference in citrate excretion (Supplemental Figure 3A). Low dose–treated mice similarly showed substantial variability and a 27% reduction in mean urinary citrate levels compared with KO+vehicle controls; however, this difference was not statistically significant (*P* = 0.6983; Supplemental Figure 3A). Importantly, altered blood or urinary citrate levels were not associated with kidney or liver toxicity as blood biochemistry markers remained within normal ranges across all groups. (Supplemental Figure 3, B–E).

### Seizure susceptibility is attenuated with AAV9/SLC13A5 treatment of Slc13a5-KO pups.

**Figure 3:**
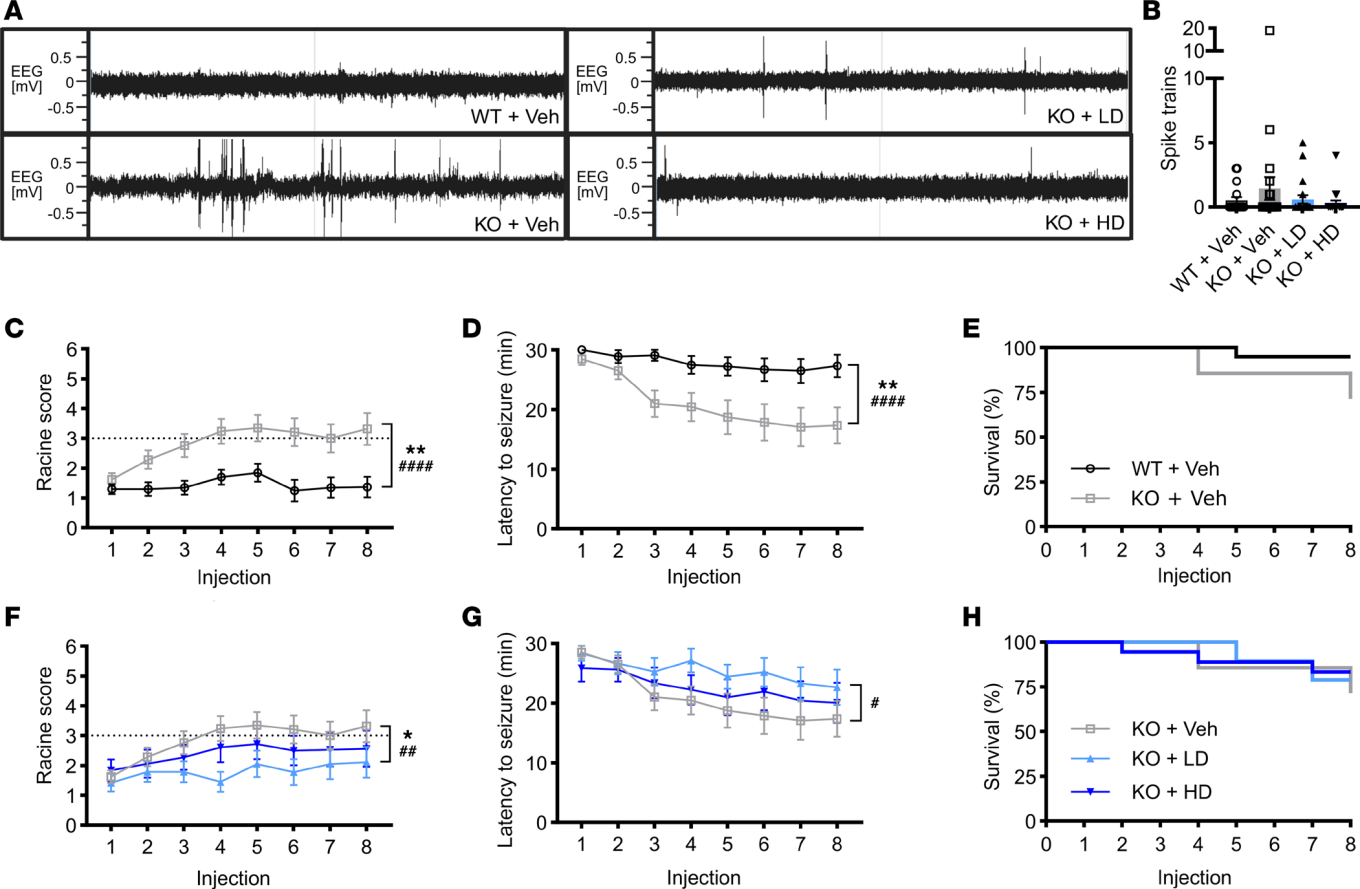
AAV9/SLC13A5 gene therapy rescues epilepsy in *Slc13a5*-KO mice treated at P10. (**A** and **B**) Surface EEG was collected at 3 months of age using a wireless telemetry implant in WT and KO mice IT-injected at P10 with vehicle or 2 × 10^11^ vg (LD) or 8 × 10^11^ vg (HD) AAV9/SLC13A5. (**A**) Representative EEG traces over a 10-minute period. (**B**) Total spike train counts over 2 recordings, 60 hours each. (**C**–**H**) Seizure susceptibility was assessed with administration of 30 mg/kg pentylenetetrazol (PTZ) every other day for 8 total injections. (**C** and **D**) Vehicle-treated *Slc13a5*-KO mice were significantly more susceptible to PTZ-induced seizures compared with WT mice, exhibiting increased seizure severity (**C**) and decreased latency to seizure (**D**). (**E**) Survival of vehicle-treated controls over the course of 8 injections. (**F**–**H**) Treatment with AAV9/SLC13A5 in KO mice reduced seizure severity (**F**) and increased seizure latency compared with KO+Vehicle mice (**G**). (**H**) Survival of PTZ study mice over the course of 8 injections. Repeated measures ANOVA as compared with KO+Veh group: genotype/treatment effect *P ≤ 0.05, **P < 0.01; genotype/treatment × PTZ injection effect ^#^*P* < 0.05, ^##^*P* < 0.01, ^####^*P* < 0.0001. *n* = 20 WT+Veh, 21 KO+Veh, 19 KO+LD, 18 KO+HD. Data shown as mean ± SEM.

Efficacy was tested, in part, by assessing epileptiform activity using wireless telemetry devices. Representative EEG traces demonstrated that ~3-month-old *Slc13a5*-KO mice had increased EEG abnormalities compared with WT mice (Figure 3A). Quantification of epileptic spike trains confirmed a small, nonsignificant increase in epileptiform discharges in control KO mice compared with WT mice (Figure 3B). In KO mice, treatment with AAV9/SLC13A5 decreased the number of spike trains in a dose-dependent manner compared with vehicle treatment (Figure 3, A and B).

*Slc13a5-*KO mice have occasional sporadic seizures and are more susceptible to seizure induction (11). In young adult KO mice we did not observe sporadic seizures, so we tested seizure susceptibility using a chronic induction paradigm using a fixed low dose (30 mg/kg) of 8 injections of pentylenetetrazol (PTZ) (29). PTZ is a well-established chemoconvulsant that induces seizures through antagonism of GABA_A_ receptor-mediated inhibitory neurotransmission, thereby lowering seizure threshold. Seizure severity was scored using a modified Racine scale, where 0 to 2 represents none to partial/focal seizures, scores of 3 to 5 are generalized seizures, and a maximal score of 6 represents prolonged tonic extensions of muscles that result in death (30). On average, WT mice failed to develop generalized seizures (score of 3 or greater) while KO mice had a significant increase in seizure severity that progressed to generalized seizures and coincided with a significant reduction in latency to seize (Figure 3, C and D). KO mice treated with low-dose AAV9/SLC13A5 had significantly decreased seizure severity and increased the latency to seize with repeat PTZ injections as compared with vehicle-treated KO mice (Figure 3, F and G). KO mice treated with high-dose AAV9/SLC13A5 had similar Racine scores and latency to seize as low dose–treated KO mice and on average failed to develop generalized seizures. Additionally, 1/20 WT+Vehicle (5%), 6/21 KO+Vehicle (29%), 4/19 KO+LD (21%), and 3/18 KO+HD (18%) mice died by the eighth injection (Figure 3, E and H). Together, these results support that early gene therapy treatment reduces epileptiform activity and protects against seizure-related death in an SLC13A5 mouse model.

### Adolescent AAV9/SLC13A5 treatment rescues EEG abnormalities and restores normal sleep.

**Figure 4:**
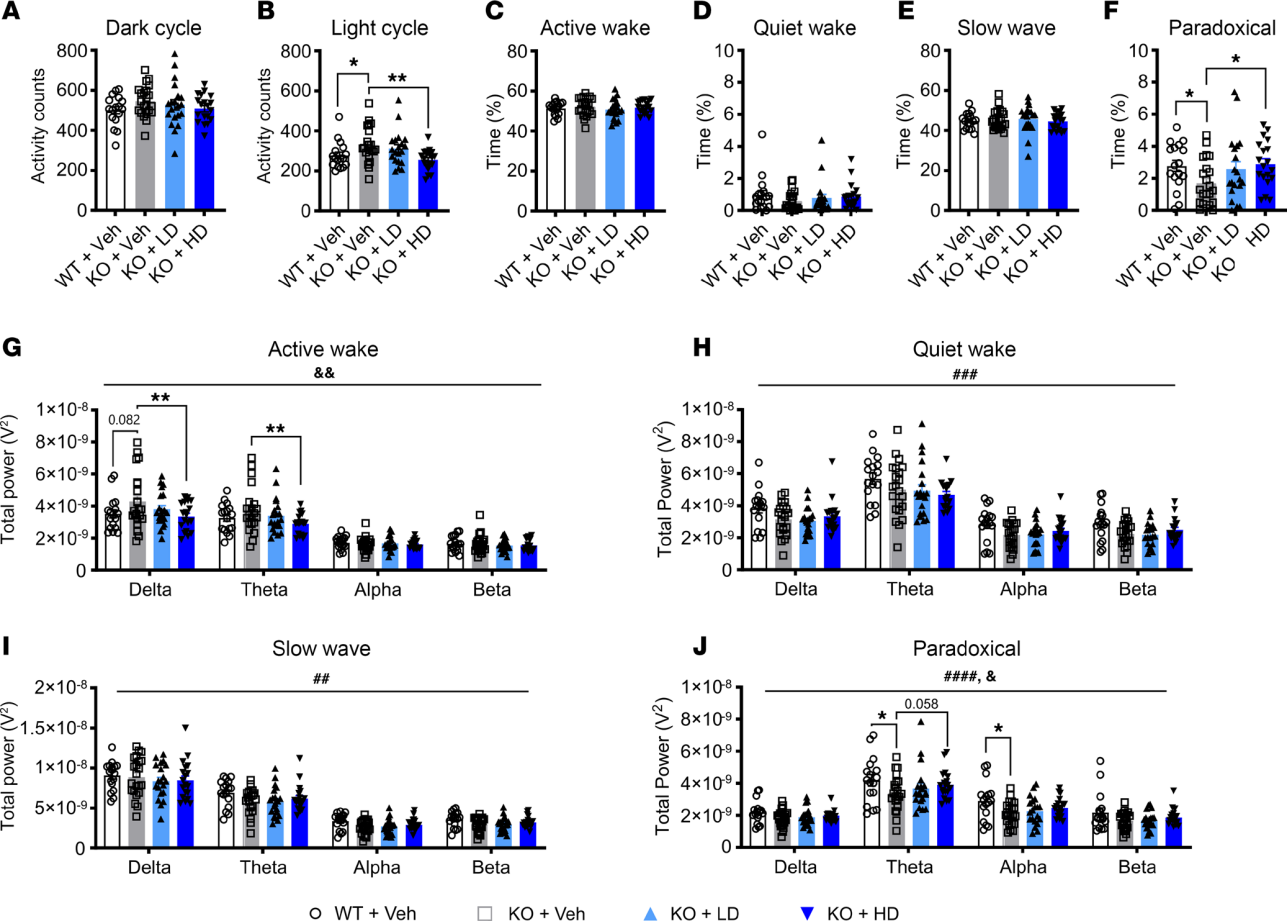
P10 IT delivery of AAV9/SLC13A5 rescues altered EEG sleep signatures in *Slc13a5*-KO mice. (**A** and **B**) Overall activity counts of WT and KO mice during the dark (**A**) and light (**B**) cycles. (**C**–**F**) Percentage of time spent in active wake (**C**), quiet wake (**D**), slow-wave sleep (**E**), and paradoxical sleep (**F**). One-way ANOVA with uncorrected Fisher’s least significant difference, *P < 0.05, **P < 0.01. (**G**–**J**) Power spectral density during active wake (**G**), quiet wake (**H**), slow-wave sleep (**I**), and paradoxical sleep (**J**), divided into delta, theta, alpha, and beta frequencies. Two-way ANOVA, genotype effect ^##^*P* < 0.01, ^###^*P* < 0.001, ^####^*P* < 0.0001, treatment effect ^&^*P* < 0.05, ^&&^*P* < 0.01; with Šídák’s multiple comparisons test (WT vs. KO) or Dunnett’s multiple comparisons test (KO vs. KO treated groups) *P < 0.05, **P < 0.01. *n* = 22 KO+Veh, 20 KO+LD, 19 KO+HD. Data shown as mean ± SEM.

Previously, we reported that the vehicle-treated young adult *Slc13a5-*KO mice have increased activity during the light cycle, when mice typically sleep; decreased paradoxical sleep; and changes in absolute power spectral density (PSD) as compared with the vehicle-treated WT mice, indicating altered sleep architecture in KO mice (2). In treated KO mice, we found that during the dark cycle, when mice are typically active, vehicle- and virus-treated KO mice had similar levels of activity (Figure 4A). During the light cycle, the abnormally high activity in untreated KO mice was normalized with the high dose (*P* = 0.0022; Figure 4B), suggesting that treated KO mice spend more time asleep. Analysis of the specific sleep stages using the EEG and electromyogram (EMG) data showed that vehicle- and vector-treated KO mice spent a similar percentage of time in active wake, quiet wake, and slow wave sleep stages (Figure 4, C–E), while the low amount of time that vehicle-treated KO mice spend in paradoxical sleep was increased with gene therapy in a dose-dependent manner (low dose *P* = 0.0853, high dose *P* = 0.0223; Figure 4F).

We then performed PSD analysis across 0–30 Hz frequencies. Frequency bands were defined as delta (0.5–4 Hz), theta (4–8 Hz), alpha (8–12 Hz), and beta (12–30 Hz). Power band analysis of treated KO mice showed a dose-dependent decrease of slow wave activity (delta and theta waves) during the active wake stage (Dunnett’s multiple comparisons test, delta: low dose *P* = 0.1530, high dose *P* = 0.0024; theta: low dose *P* = 0.3327, high dose *P* = 0.0060). Previously, we reported that the absolute PSD during paradoxical sleep was significantly altered in vehicle-treated KO mice as compared with vehicle-treated WT mice, with theta and alpha power bands being significantly decreased in KO mice (2). In gene therapy–treated mice, we found an increase of absolute PSDs during paradoxical sleep (2-way ANOVA, treatment effect *F*_2, 232_ = 3.574, *P* = 0.0296), with a dose-dependent increase of theta power waves (Dunnett’s multiple comparisons test, low dose: *P* = 0.3573, high dose: *P* = 0.0582), while alpha waves were similar among all treatment groups (Figure 4, G–J). Together these results support that AAV9/SLC13A5 treatment in P10 KO mice achieves a dose-dependent rescue of sleep dysfunction.

### Gene therapy treatment results in a sustained functional NaCT.

Given that AAV9/SLC13A5 treatment confers neurological benefit, we next examined potential factors that may contribute to this effect. In addition to increased blood citrate levels, *Slc13a5-*KO mice also exhibit elevated citrate levels in the CSF, and extracellular citrate has been hypothesized to contribute to neuronal hyperexcitability in this disorder (8, 11, 31). To determine if AAV9/SLC13A5 gene replacement therapy results in a sustained decrease of CSF citrate levels, a second cohort of mice was injected at P10 with 2 × 10^11^ vg (low dose), and CSF was collected at 6–7 months postinjection (Supplemental Figure 4A). LC-MS analysis confirmed that vehicle-treated *Slc13a5-*KO mice had significantly higher CSF citrate levels compared with vehicle-treated WT mice (Supplemental Figure 4B). Gene therapy treatment decreased CSF citrate levels to 81% ± 3.9% of WT levels (Supplemental Figure 4B).

To further characterize transgene expression across the brain, we examined cellular transduction patterns. Costaining of NaCT with cell-specific markers confirmed that, in agreement with prior characterizations of AAV9 tropism, the AAV9/SLC13A5 vector transduced both neurons and glia (Supplemental Figure 4, C–F) (32, 33). Importantly, this NaCT expression was observed in brains assessed up to 7 months postinjection, indicating sustained transgene expression. In KO + low-dose–treated mice, NaCT^+^ cells were more frequently neuronal (MAP2^+^) than astrocytic (S100B^+^). For example, within the brain stem, 16% of MAP2^+^ cells were NaCT^+^ compared with 6% of S100B^+^ cells, which was similar across other regions analyzed, including the hippocampus and cortex (Supplemental Figure 4, C–F). Collectively, these findings support that AAV9/SLC13A5 gene therapy results in sustained, functional NaCT expression with predominant neuronal transduction.

### ICM delivery of AAV9/SLC13A5 in adult KO mice results in greater brain NaCT expression than IT delivery.

**Figure 5:**
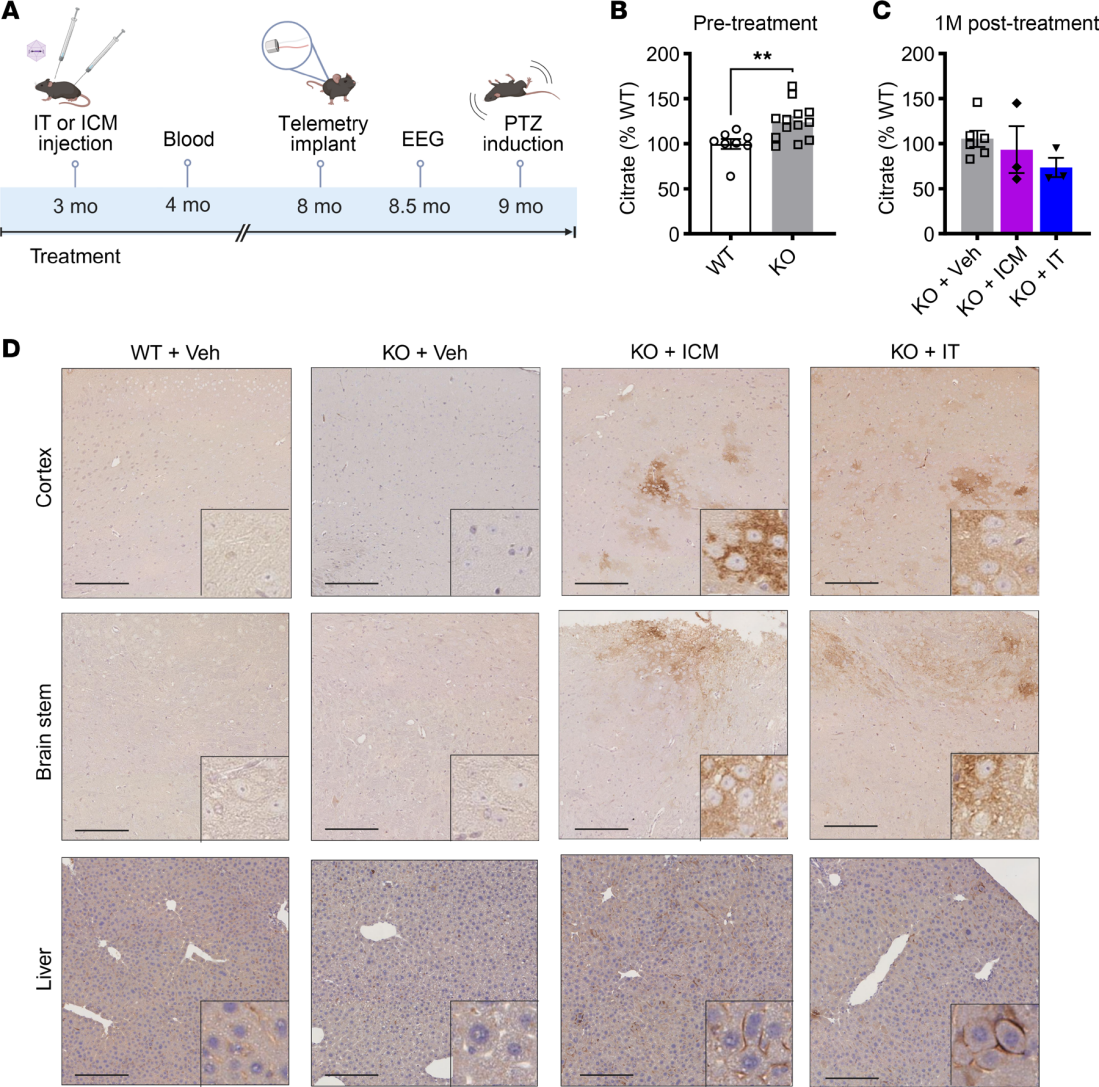
ICM delivery of AAV9/SLC13A5 in adult KO mice results in greater brain NaCT expression compared with IT delivery. (**A**) Schematic of the efficacy study in *Slc13a5*-KO mice ICM- or IT-injected at 3 months with vehicle or 8 × 10^11^ vg AAV9/SLC13A5. (**B** and **C**) GC-MS analysis of plasma citrate, relative to WT+Vehicle controls. (**B**) Pretreatment WT and KO controls. *n* = 8 WT, 13 KO. Student’s unpaired *t* test, **P < 0.01. (**C**) Vehicle- AAV9/SLC13A5 ICM–, and IT-treated KO mice 1 mo posttreatment. *n* = 6 KO+Veh, 3 KO+ICM, 3 KO+IT. (**D**) Representative images of mouse brain collected 6 months posttreatment and stained with SLC13A5/NaCT antibody in the cortex (top), brain stem (middle), and liver (bottom). Scale bar: 200 μm.

Having found that gene therapy treatment in developing mice provided benefit, we then asked if treatment in adult mice, later in the disease course and after brain development is complete, is beneficial. Our prior work showed that IT lumbar puncture injection of AAV9 in adult mice results in significantly decreased brain transduction as compared with mice injected at P10 (33). To address this, we evaluated delivery route as a strategy to enhance CNS exposure in adult mice. ICM delivery has been shown by our group and others to achieve greater brain transduction than IT delivery in adult mice (32). To test this, young adult (3 mo) *Slc13a5-*KO and WT mice were IT- or ICM-treated injected with vehicle or AAV9/SLC13A5 and were followed for approximately 6 months postinjection (Figure 5A) (32). Adult treatment was well tolerated, with no adverse effects observed across all the animals treated as determined by body weight gain/maintenance, survival, and general activity (Supplemental Figure 5).

Blood was assessed for citrate from a subset of WT and KO mice prior to AAV injection and 1 month postinjection. In agreement with our vehicle-treated mice from our prior study (Figure 2), we found that untreated KO mice had significantly increased blood citrate levels compared with untreated WT mice (Figure 5B). Treatment with AAV9/SLC13A5 decreased plasma citrate levels in KO mice to 93.3% ± 26.1% of WT levels via ICM administration and to 73.7% ± 10.7% via the IT route (Figure 5C). We also assessed SLC13A5 transgene expression in the brain by staining for NaCT protein. In AAV9/SLC13A5-treated mice, there was widespread expression of the SLC13A5 protein throughout the brain and liver, with higher expression achieved across the brain with ICM delivery as compared with IT delivery (Figure 5D and Supplemental Figure 6). Compared with mice treated with the same dose and via the same route at P10 (Figure 2 and Supplemental Figure 1), KO IT-injected at 3 mo had relatively lower NaCT levels throughout the brain.

### AAV9/SLC13A5 treatment of adult KO mice rescues epileptic phenotypes.

**Figure 6:**
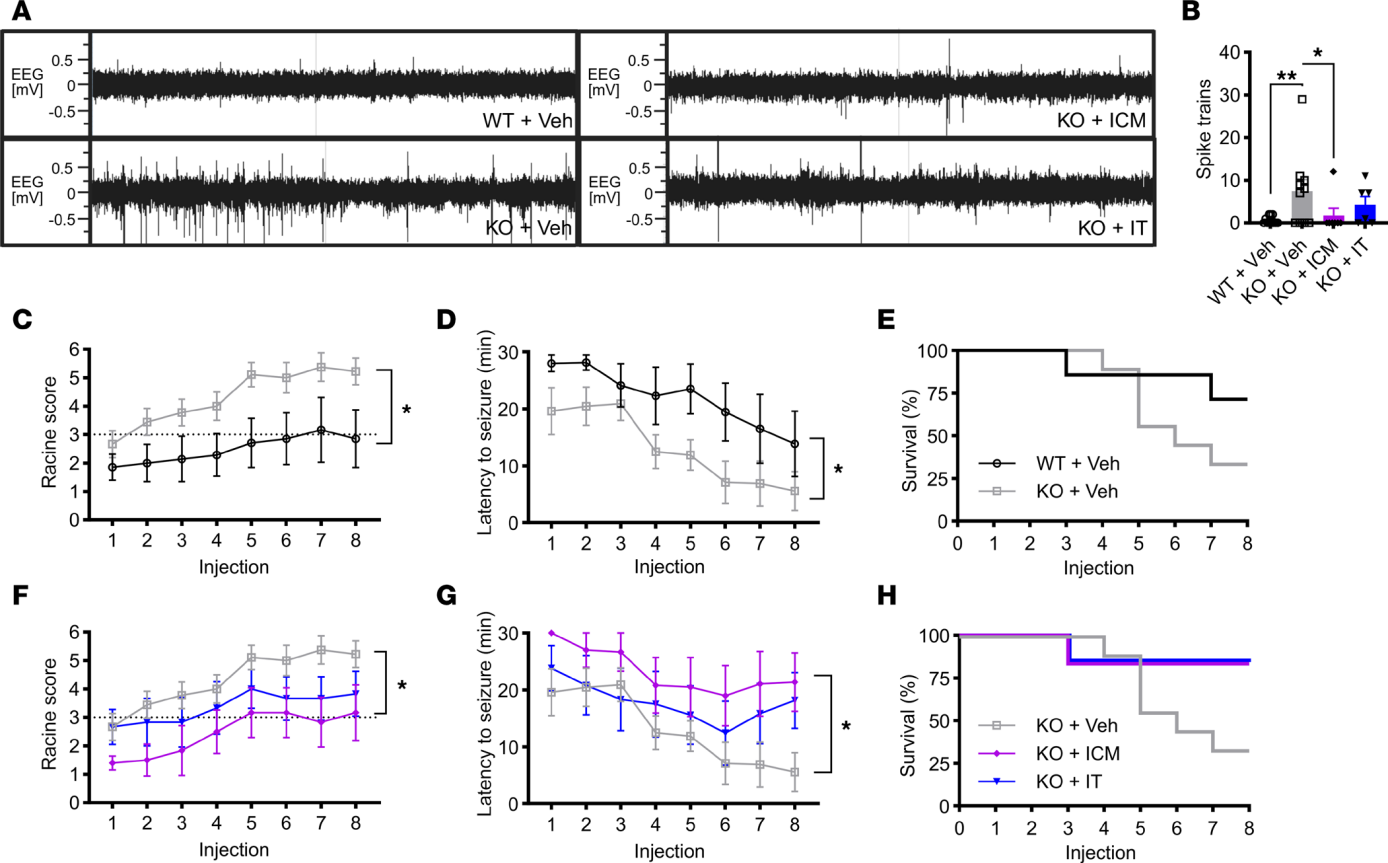
ICM delivery of AAV9/SLC13A5 has greater rescue of epileptic phenotypes compared with IT delivery in adult *Slc13a5*-KO mice. (**A** and **B**) Surface EEG collected from WT and KO mice injected at 3 months via ICM or IT with vehicle or 8 × 10^11^ vg AAV9/SLC13A5. (**A**) Representative EEG traces over a 10-minute period. (**B**) KO mice exhibited elevated spike train counts that were reduced with gene therapy. One-way ANOVA, *P < 0.05, **P < 0.01. (**C**–**H**) Seizure susceptibility was assessed with administration of 30 mg/kg PTZ every other day for 8 total injections. (**C**–**E**) Vehicle-treated *Slc13a5*-KO mice were significantly more susceptible to PTZ-induced seizures compared with WT mice, exhibiting increased seizure severity (**C**), decreased latency to seizure (**D**), and increased incidence of seizure-induced death (**E**). (**F**–**H**) Seizure susceptibility of AAV9/SLC13A5-treated mice were compared with KO+Vehicle mice. ICM delivery resulted in significantly decreased seizure severity (**F**) and decreased latency to seizure (**G**). (**H**) ICM and IT delivery of AAV9/SLC13A5 reduced seizure-induced death. Repeated measures 2-way ANOVA as compared with KO+Veh group: genotype/treatment effect *P ≤ 0.05. *n* = 7 WT+Veh, 9 KO+Veh, 6 KO+ICM, 6 KO+IT. Data shown as mean ± SEM.

Mice were implanted with telemetry devices at ~5 months postinjection, and EEG, EMG, and activity data were recorded over two 60-hour recording periods. In WT mice, EEG brain activity was normal while KO mice treated with vehicle had increased epileptiform activity (Figure 6A). ICM administration of AAV9/SLC13A5 normalized brain activity of KO mice to WT levels and to a lesser extent with IT administration (Figure 6A). Quantification of spike trains showed that compared with WT mice, KO mice had significantly increased number of spike trains and that treatment with AAV9/SLC13A5 resulted in a significant decrease in the number of spike trains with ICM administration and to a lesser extent with IT administration (Figure 6B).

Mice were then assessed for seizure susceptibility using our PTZ kindling paradigm described above. At 9 months of age, WT mice had an average Racine Score of 2.9 ± 1.0 by injection 8. In contrast, control KO mice were significantly more susceptible to seizure induction and had a mean Racine Score of 5.2 ± 0.5 by injection 8 and a significantly reduced latency to seize as compared with WT mice (Figure 6, C and D). The increased seizure susceptibility resulted in early lethality in 2/8 WT+Vehicle mice (25%) and 6/9 KO+Vehicle mice (67%) (Figure 6E). Treatment of KO mice with AAV9/SLC13A5 decreased seizure severity with a significant improvement when delivered via the ICM route and to a lesser extent via the IT route as compared with vehicle-treated KO mice (Figure 6F). By injection 8, KO+ICM mice and KO+IT mice had an average Racine Score of 3.2 ± 1.0 and 3.8 ± 0.8, respectively. AAV9/SLC13A5-treated KO mice also had increased latency to seizure onset as compared with KO control mice (Figure 6G). By decreasing seizure susceptibility, seizure-related deaths were decreased in treated KO mice, where only 1/6 KO+ICM (17%) and 1/6 KO+IT (17%) died by the eighth injection (Figure 6H). Together, these results support that AAV9/SLC13A5 administration in young adult mice restored resistance to the onset of seizures and reduced seizure severity.

### Adult AAV9/SLC13A5 treatment does not alter general activity levels.

Analysis of telemetry implant data showed that at 8 months of age, conscious, freely moving KO mice had similar activity levels within their home cages during the light and dark periods as compared to WT mice (Supplemental Figure 7). When treated mice were assessed, we found that injection of AAV9/SLC13A5 by either ICM or IT delivery route did not significantly alter the activity of older KO mice (Supplemental Figure 7).

### Sleep dysfunction and abnormal electrophysiologic power spectrum are ameliorated in Slc13a5-KO mice when treated as adults.

**Figure 7:**
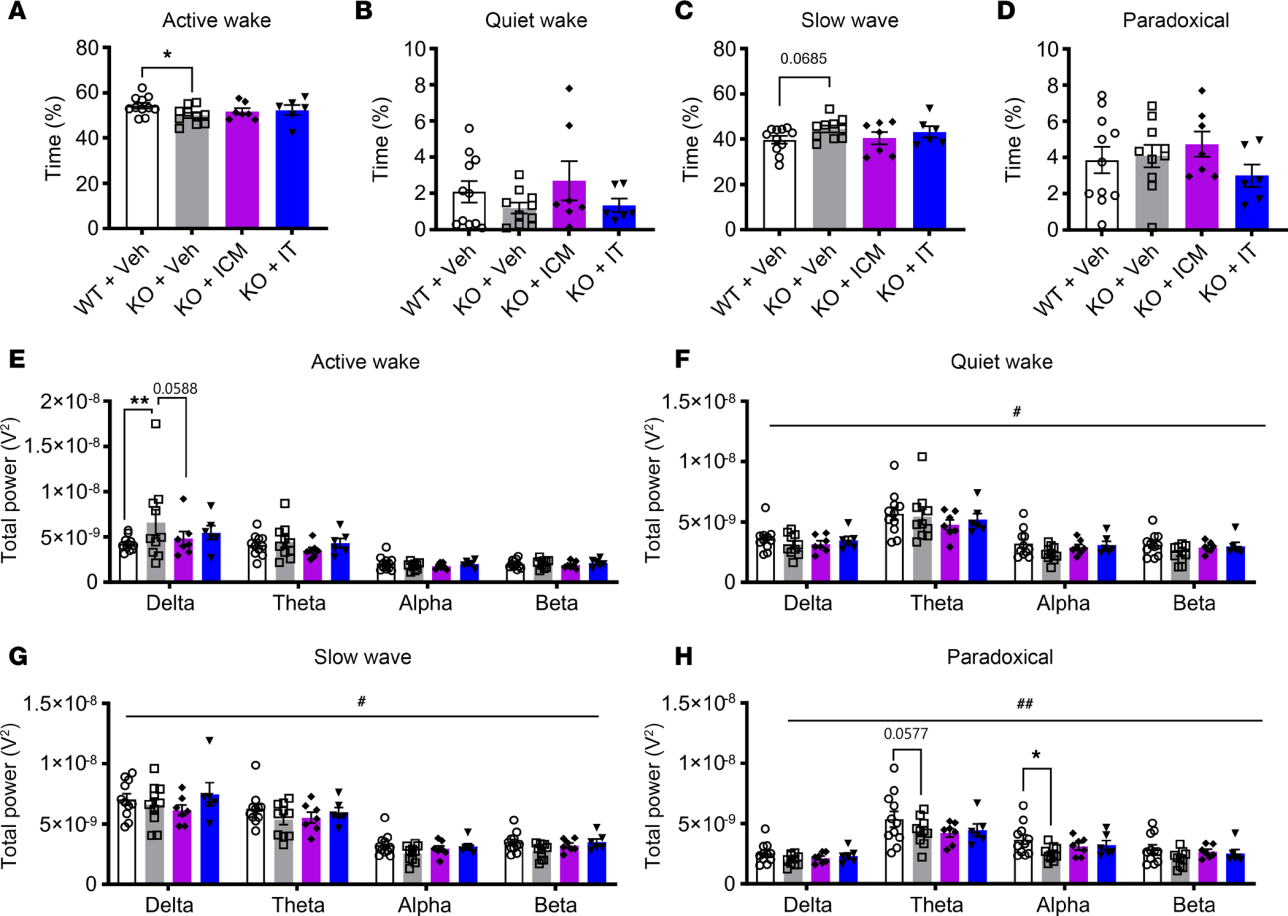
AAV9/SLC13A5 treatment in adult *Slc13a5*-KO mice ameliorates sleep abnormalities. (**A**–**D**) Assessments of percentage of time spent in active wake (**A**), quiet wake (**B**), slow-wave sleep (**C**), and paradoxical sleep (**D**) in vehicle- and AAV9/SLC13A5-treated WT and KO mice. One-way ANOVA with uncorrected Fisher’s LSD, *P < 0.05. (**E**–**H**) Power spectral density during active wake (**E**), quiet wake (**F**), slow-wave sleep (**G**), and paradoxical sleep (**H**), divided into delta, theta, alpha, and beta frequencies in WT Vehicle, KO Vehicle, and AAV9/SLC13A5 ICM- and IT-treated mice. Two-way ANOVA, genotype effect ^#^*P* < 0.05, ^##^*P* < 0.01; with uncorrected Fisher’s LSD, *P < 0.05, **P < 0.01. *n* = 11 WT+Veh, 10 KO+Veh, 7 KO+ICM, 6 KO+IT. Data shown as mean ± SEM.

At 8 months of age, KO mice spent significantly less time in the active wake phase and more time in the slow wave sleep phase as compared with WT mice (Figure 7, A–D). This supports that older adult *Slc13a5-*KO mice have altered sleep physiology, although in contrast with young adult animals (2), sleep dysfunction was characterized by less time spent awake and more time spent asleep. When KO mice were treated with AAV9/SLC13A5, there was a trending increase in the amount of time spent in active wake and decreased time spent sleeping as compared with vehicle-treated KO mice, with the greatest rescue seen in mice that were ICM-treated versus IT-treated (Figure 7, A–D).

The power spectra within the specific sleep/wake stages were then assessed. We found that delta waves significantly increased in KO mice during the active wake period as compared with WT mice, which decreased with treatment of AAV9/SLC13A5, with a stronger improvement observed in the ICM route of administration (Figure 7E). In contrast, during quiet wake, slow wave sleep, and paradoxical sleep, KO mice had an overall decrease of absolute PSD across all frequencies (including delta) as compared with WT mice (Figure 7, F–H). In agreement with our prior PSD analysis of 4-month-old KO mice, theta and alpha waves in paradoxical sleep were decreased in KO mice compared with WT (Figure 7H) (2). Treatment with AAV9/SLC13A5 resulted in a general increase of the absolute spectral power during quiet wake, slow wave sleep, and paradoxical sleep (Figure 7, F–H). Additionally, during paradoxical sleep, there was a trend of increased alpha activity in ICM and IT AAV9/SLC13A5-treated KO mice as compared with vehicle-treated KO mice (Figure 7H). Together, these results support that older adult *Slc13a5-*KO mice have sleep disturbances that result in increased slow wave power during wake periods, which can be ameliorated with AAV9/SLC13A5 treatment.

### Vector brain biodistribution was dose dependent when administered in young mice and route dependent in adult mice.

**Figure 8:**
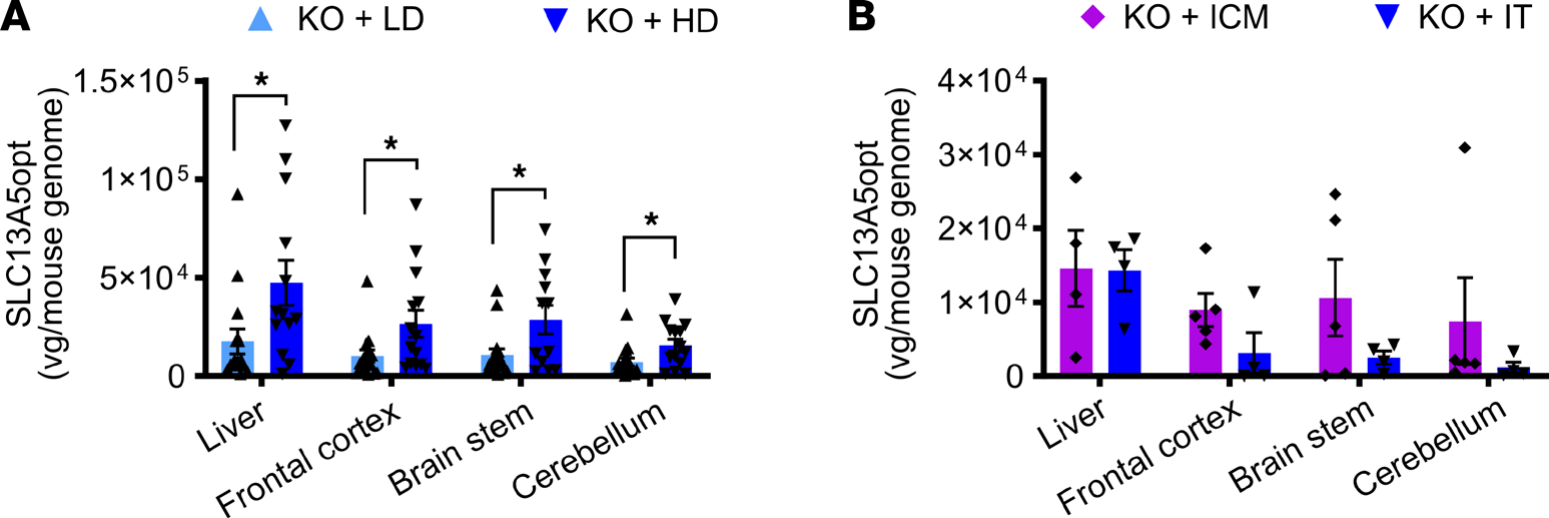
Biodistribution of AAV9/SLC13A5 following CSF delivery in *Slc13a5*-KO mice. (**A** and **B**) Copies of AAV9/SLC13A5 per host genome in the liver, frontal cortex, brain stem, and cerebellum in mice dosed at P10 (**A**) and 3 months (**B**). Student’s unpaired *t* test, *P < 0.05. *n* = 15 KO+LD, 14 KO+HD, 5 KO+ICM, 4 KO+IT. Data shown as mean ± SEM.

To assess vector distribution with the different ages of injection, routes of delivery, and doses, liver and brain (frontal cortex, brain stem, cerebellum) were assessed via droplet digital PCR (ddPCR) for SLC13A5 transgene. Overall, *Slc13a5-*KO animals dosed at P10 with the high dose had a significantly greater abundance of AAV9/SLC13A5 vg/μg host genomic DNA when compared with those dosed with the low dose (Figure 8A). As expected for IT delivery of AAV9, the liver had a greater amount of vector than the brain (19, 32). KO animals dosed at 3 mo by ICM administration had a larger abundance of AAV9/SLC13A5 in the brain (cerebellum: 7.5 × 10^3^ ± 5.9 × 10^3^ vg/mouse genome) compared with animals dosed by IT administration (cerebellum: 1.2 × 10^3^ ± 7.4 × 10^2^ vg/mouse genome), while the amount of vector present in the liver was comparable (ICM: 1.5 × 10^4^ ± 5.2 × 10^3^ vg/mouse genome; IT: 1.4 × 10^4^ ± 2.8 × 10^3^ vg/mouse genome; Figure 8B). In agreement with our prior work in WT mice (33), IT delivery of AAV9 in KO mice at P10 resulted in markedly higher brain transduction (cerebellum: 15.7 × 10^3^ ± 3.1 × 10^3^ vg/mouse genome) as compared with KO mice IT injected with the same dose at 3 mo (cerebellum: 1.2 × 10^3^ ± 7.4 × 10^2^ vg/mouse genome). Together, the biodistribution data support that CSF delivery of AAV9/SLC13A5 via IT lumbar puncture or ICM injection efficiently transduces target tissues important for SLC13A5 citrate transporter disorder, with greater distribution achieved with administration in younger animals versus older animals.

## Discussion

Patients with SLC13A5 citrate transporter disorder develop seizures within the first days of life and have lifelong epilepsy, neurocognitive impairments, a profound movement disorder, and limited verbal communication abilities that impact patient and family quality of life (4). Currently, there are no curative treatments that address the underlying disease cause; many patients require multiple antiseizure medications simultaneously, and antiseizure medications are not effective in all patients. Furthermore, patients require multiple additional medications, therapies, and support to address additional symptoms. We have developed a gene replacement approach for SLC13A5 deficiency that could have a profound impact on patients, potentially even into adulthood. Our preclinical studies showed that when packaged in AAV9 and delivered to *Slc13a5-*KO pups or young adults, typical brain activity was restored, sleeping deficits were improved, and seizure resistance was increased. Results from these studies support that onetime CSF administration of AAV9/SLC13A5 is effective and sufficient in improving SLC13A5 deficiency–related dysfunctions.

SLC13A5-encoded NaCT is most highly expressed in liver, brain, bone, and reproductive organs and is expressed in other organs to a lower extent (34–36). While NaCT plays a role in citrate uptake and energy metabolism in the liver, the impact of the liver phenotype on disease is unknown. The neurological phenotype is most prominent in SLC13A5 citrate transporter disorder (8), and the greatest clinical benefit is likely to be achieved using strategies aimed at restoring SLC13A5 expression in the brain. We used IT or ICM administration of AAV9, as it results in higher transduction in the CNS and relatively lower transduction of peripheral tissues as compared with systemic delivery (18, 19). Analysis of vector biodistribution and expression in mice showed that P10 IT delivery resulted in widespread vector distribution and SLC13A5 expression throughout the brain. As expected, treatment with the high dose resulted in increased vector transduction and expression as compared with the low dose. Biodistribution analysis of tissues from adult-treated mice showed that IT and ICM administration of AAV9 resulted in similar liver transduction by both routes while ICM delivery resulted in greater brain expression as compared with IT injection. ICM delivery also provided the greatest rescue of epileptiform activity and restoration of seizure resistance, which supports brain NaCT deficiency as the main driver of disease. This also supports a lesser role for deficient liver NaCT expression for the neurological phenotypes we assessed. As AAV9 transduces both neurons and glia (27, 32, 33), it is unclear whether rescue of both cell types is necessary to ameliorate epileptiform activity or if restoring neuronal or glial NaCT function alone would be sufficient.

Administration of AAV9/SLC13A5 early in brain development provided therapeutic benefit to *Slc13a5-*KO mice as assessed by citrate levels, brain activity, sleep behavior, and susceptibility to seizures and seizure-related death. Comparison of the low dose– and high dose–treated P10 mice showed benefit at the low dose that was even more beneficial with the high dose in most outcomes assessed, including EEG activity and sleep. Importantly, we also found that AAV9/SLC13A5 administration to adult mice, after neuronal development, still had substantial benefit. The potential for gene therapy to provide benefit when administered into young adult patients with SLC13A5 is strengthened by the fact that although patients plateau in their development, there is no evidence of regression (1). Patients with SLC13A5 disorder have structurally normal brains, and while epileptiform activity is prominent, EEGs are typically not disorganized (5, 37). This indicates that brain architecture is largely intact in patients and suggests a wide age range for when gene therapy can be administered to restore functional NaCT and normalize brain activity.

In agreement with prior work from our group and others, AAV9 transduced both neuronal and astrocytic populations (32, 33). In earlier studies, we reported that intrathecal delivery of AAV9 at P10 resulted in proportionally higher astrocyte transduction relative to neurons, whereas delivery at later ages (up to 3 months) led to relatively greater neuronal targeting because of a decline in astrocyte transduction with age (33). In the present study, mice were injected at P10 but analyzed at a substantially later time point (6–7 months postinjection). At this later time point, we observed a higher proportion of NaCT-positive neurons compared with astrocytes. This shift may reflect differences in the durability of transgene expression across cell types over time, as prior analyses were performed within weeks of injection, whereas the current study assessed sustained expression months later. Alternatively, differences in ubiquitous promoter usage between studies (Cbh versus UsP) may contribute to the observed cell type distribution. At present, the relative contributions of neuronal versus astrocytic NaCT deficiency to the neurological manifestations of SLC13A5 deficiency remain unclear. However, the predominance of neuronal transduction at later time points, together with the observed long-term neurological benefits, raises the possibility that neuronal restoration of NaCT may be a key contributor to therapeutic efficacy. Future studies employing cell type–specific modulation of NaCT expression will be important to delineate the cellular drivers of disease pathophysiology and to inform optimization of gene therapy strategies for this disorder.

To assess seizure susceptibility and therapeutic efficacy in vivo, we used PTZ as a standardized chemoconvulsant challenge. Although PTZ does not model the primary metabolic defect underlying SLC13A5 citrate transporter disorder, it provides a sensitive and widely used assay of neuronal network excitability by antagonizing GABA_A_ receptor–mediated inhibition (29). Epilepsy in SLC13A5 deficiency is characterized by early-onset, treatment-resistant seizures and heightened sensitivity to diverse triggers, consistent with a reduced seizure threshold rather than a single ictogenic mechanism. Disrupted neuronal citrate transport is thought to impair energy metabolism, redox balance, and neurotransmitter homeostasis, creating a permissive state of hyperexcitability (38). In this context, PTZ challenge served as a functional readout of circuit stability and inhibitory-excitatory balance. The observed improvement in PTZ seizure resistance following gene replacement supports a biologically meaningful restoration of neuronal excitability, even though PTZ itself is not a disease-specific seizure trigger. Future studies incorporating spontaneous seizure monitoring and metabolic stress paradigms will further refine the alignment between preclinical seizure assays and clinical epilepsy phenotypes in SLC13A5 citrate transporter disorder.

Surprisingly in our PTZ kindling paradigm, low-dose AAV9/SLC13A5 administered at P10 provided similar benefit as high dose. This may indicate a plateauing therapeutic effect with vector administration at P10. In transgenic mice, overexpression of SLC13A5 in a subpopulation of neurons (CAMKIIa^+^) beginning during embryonic development was reported to cause autistic-like and jumping behaviors, and altered white matter integrity and synaptic plasticity, with aberrant synaptic structure and function (39). As S*lc13a5* gene expression in the rat cerebral cortex is low at birth and increases in the days following birth (40), the overexpression studies raise a concern that high levels of NaCT from vector expression during the prenatal or immediate post-neonatal period may impact proper brain development in rodents. While this could be contributing to the lack of further rescue of seizure susceptibility in the high-dose P10 treated cohort, there was greater benefit with the high dose than low dose in all other metrics we measured, including reducing epileptic discharges, normalizing brain waves, and ameliorating sleep deficits. Additionally, high-dose AAV9/SLC13A5 treatment was well tolerated as mice had normal body weight, general activity, neurological function, and survival as compared with WT mice.

Epileptic phenotypes were notably different between younger and older adult mice, which can be reflective of alterations in inflammation, network function, and vulnerability to insult with aging. PTZ-induced seizures in mice have been reported to worsen with age (41, 42), which was recapitulated within our findings, where WT mice tested at 4 months of age had an average Racine Score of 1.4 (Figure 3C) while WT mice tested at 9 months of age had an average Racine Score of 2.9 (Figure 6C). Further, we did not detect spontaneous seizures during the 60-hour recording periods in young adult KO mice while spontaneous seizures occurred in a few older adult vehicle-treated KO mice but not AAV-treated mice. Similarly, the sleep phenotype varied with age. Three-month-old KO mice had increased movement during the typical sleep period for rodents, and this coincided with decreased time spent during paradoxical sleep (2). In contrast, 8-month-old KO mice spent significantly less time in the active wake stage and more time in the slow wave sleep as compared with WT littermates, which could indicate that with aging abnormal excess sleepiness becomes a defining feature of *Slc13a5-*KO mice. Aging in mice and humans has also been shown to disrupt the ability to sustain sleep/wake states, particularly in wakefulness and the transition to non-REM sleep (43, 44).

A consistent feature of young and older adult *Slc13a5-*KO mice was lower theta and alpha waves during paradoxical sleep. AAV9/SLC13A5 treatment had a greater effect when treated at P10, where there were a significant decrease in hyperactivity during the sleep cycles (Figure 4B) and an increase in total paradoxical sleep (Figure 4F) and which were similar to WT levels (2). AAV9/SLC13A5 treatment at 3 mo had less effect in normalizing theta and alpha waves in KO mice (Figure 7, E–H). This may be due to group sizes and being underpowered or indicates a neurodevelopmental feature of the disorder that is not rescued with adult vector administration. Interestingly, 8-month-old vehicle-treated KO animals had significantly increased delta wave power during the active wake period as compared with WT littermates (Figure 7E). Delta brain waves are typically a hallmark of sleep and should not be excessive during an awake state. Remarkably, gene therapy administration in adult animals was effective at decreasing delta activity during the active wake state, with greater normalization achieved with higher brain transduction (Figure 7E).

NaCT is responsible for transporting citrate from the extracellular fluid into the cell. In the absence of functional NaCT, citrate levels are distinctly increased in both the blood and CSF of patients and mice (7, 8, 10). While elevated extracellular citrate levels are a marker for impaired NaCT activity, they may not directly correlate with disease outcome (9). Our *Slc13a5-*KO mice had significantly increased citrate in the blood and CSF, where citrate was ~20% more in the blood and ~50% more in the CSF as compared with WT levels. Treatment at P10 or at 3 mo significantly decreased both blood and CSF citrate levels, indicating functional NaCT activity. In treated mice, citrate levels decreased to as low as 65% of normal WT levels with the high vector dose at P10. Importantly, this reduction was well tolerated, with no gross abnormalities observed at necropsy in any tissue, including kidneys.

The clinical implications of reduced circulating citrate in the context of restored citrate uptake remain incompletely understood. Citrate not taken up by tissues is filtered by the kidneys and excreted in the urine, where it plays a critical role in maintaining acid-base homeostasis and in preventing calcium nephrolithiasis, or formation of kidney stones (45). While urinary citrate concentrations were decreased in a subset of treated animals, most values remained within the range of variability observed in control groups and were not associated with renal or hepatic dysfunction as assessed by blood biochemistry. Together, these findings suggest that AAV9/SLC13A5 treatment does not induce clinically concerning alterations in urinary citrate handling or renal function within the time frame evaluated.

While adverse effects related to prolonged or excessive NaCT expression were not evaluated in the present study, these findings underscore the importance of extended monitoring of efficacy, immunogenicity, and neurodevelopmental outcomes. Consideration of NaCT expression and potential effects in peripheral tissues is important for anticipating off-target effects, renal outcomes, and broader phenotypic correction. To address these considerations, long-term toxicology and biodistribution studies across multiple species have been completed and will be reported separately.

Compared with the clinical phenotype observed in patients with SLC13A5 citrate transporter disorder, *Slc13a5-*KO mice have a relatively mild phenotype and lack substantial motor and cognitive deficits that are important clinical features (46, 47), and a limitation of our studies was our inability to determine if gene therapy can improve motor and cognitive functions. These differences between mice and humans may indicate underlying biochemical (e.g., different *K_M_*) or physiological differences in NaCT function between species (46, 47). Currently, there is not an animal model that robustly models all aspects of the human disorder, and the KO mice were the best option available for testing our gene therapy.

We were able to use *Slc13a5-*KO mice to confirm NaCT protein expression following AAV9/SLC13A5 therapy, transporter function, and vector distribution within the brain and throughout the body. Additionally, changes in citrate levels, brain activity, sleep, and seizure susceptibility were used to determine if AAV9/SLC13A5 was beneficial and informed critical variables in therapy, such as dosing, route of administration, and age of treatment. For clinical translation, follow-up studies are needed to determine long-term effects of NaCT overexpression and the distribution profile in nontarget tissues.

Since most pathogenic variants identified in individuals affected with SLC13A5 citrate transporter disorder are missense mutations that produce partially functional or misfolded proteins, future work should prioritize assessing safety and efficacy within a model carrying patient-relevant point mutations (48). It remains unclear whether a knockin model would more accurately reflect patient phenotypes; however, it is possible this could yield phenotypes distinct from complete KO. While the presence of mutant protein may affect cellular localization or introduce potential competition with AAV9-derived protein, these effects are expected to be limited given the relatively low endogenous expression compared with the high vector genome load observed following gene therapy delivery.

These results support that AAV9/SLC13A5 may be beneficial to patients with SLC13A5 citrate transporter disorder in restoring functional NaCT, restoring typical brain activity, normalizing sleep, and reducing epilepsy. Analysis of citrate in blood or CSF may serve as a biochemical marker of functional NaCT being restored. Overall, our findings support further development and assessment of AAV9/SLC13A5 safety and efficacy.

## Methods

### Sex as a biological variable.

Our study examined male and female animals, and similar findings are reported for both sexes.

### Animals.

C57BL/6J *Slc13a5*-KO mice were a gift from Rafael De Cabo from the National Institute on Aging, Bethesda, Maryland, USA, and were generated as previously described (10). Mice were bred in University of Texas Southwestern (UTSW) animal barrier facilities under controlled environmental conditions, with 12-hour light/dark cycles, and with access to commercial 2916 chow and chlorinated, reverse osmosis water ad libitum. The *Slc13a5*-KO line was maintained using C57BL/6J mice from Jackson Laboratory (strain 000664). Breeding pairs of either heterozygous males and females or homozygous males and heterozygous females were used to generate *Slc13a5*-KO mice with litter-, sex-, and age-matched controls.

### Viral vectors.

We designed and developed the UsP-hSLC13A5opt-SpA plasmids (Figure 1A). Self-complementary AAV9 vector was produced using methods developed by the University of North Carolina Vector Core facility. The purified AAV was dialyzed in PBS supplemented with 5% d-sorbitol and an additional 212 mM NaCl (350 mM NaCl total). Vector was titered by qPCR and confirmed by polyacrylamide gel electrophoresis and silver stain. Three vector lots were used across studies. See Supplemental Figure 8 for the certificate of analysis for each lot.

### Virus delivery.

SLC13A5-homozygous KO and WT mice were treated at P10 or 3 mo. At P10, mice received an IT lumbar puncture injection of vehicle or 2 × 10^11^ vg (low dose) or 8 × 10^11^ vg (high dose) of AAV9/SLC13A5. At 3 mo, mice were injected with vehicle or 8 × 10^11^ vg of AAV9/SLC13A5 via ICM or IT injection. Cage mates consisted of both vehicle- and vector-treated mice. On the day of injection, cage mates were randomly assigned to treatment groups, which were balanced for sex. For the IT injection procedure, mice were unanesthetized. Mice were held securely near the pelvic girdle, and using a 30-gauge needle (Hamilton, catalog 7803-07) with a Hamilton syringe (VWR, catalog 89210-094), 5 μL of vector solution was slowly injected into the CSF space between the L5 and L6 vertebrae. The needle was held in place for 2 seconds postinjection, then rotated, and withdrawn. For the ICM injection procedure, mice were anesthetized using an isoflurane vaporizer. Following induction of anesthesia and surgical prep, a small incision (5–10 mm) was made in the skin at the base of the skull. Lidocaine 2% (~1–2 drops) was topically applied to the incision site. Using a 30-gauge needle (Hamilton, catalog 7803-07) with a Hamilton syringe (VWR, catalog 89210-094), 10 μL of vector solution was injected into the cisterna magna. The needle was then removed and the skin closed. Mouse body weight and survival were recorded from the date of injection**.** Animals were then followed up to 6 months postinjection and assessed for brain electrophysiologic activity, seizure susceptibility, blood citrate levels, vector biodistribution, and NaCT expression. See Supplemental Table 1 for injection cohorts, study numbers, and treatments.

### Immunocytochemistry.

HEK293T cells were purchased from ATCC and routinely tested for mycoplasma contamination. Cells were maintained in DMEM with 10% fetal bovine serum, 1% glutamax, and 1% Pen/Strep. Sterile 12 mm coverslips were coated with 50 μg/mL of poly-d-lysine in borate buffer. HEK293T cells were plated on coverslips in a 24-well culture plate at a density of 5.0 × 10^4^ cells per well. At 24 hours after plating, cells were transfected with 0.5 μg GFP plasmid alone or in combination with 0.5 μg plasmid AAV/SLC13A5opt using Lipofectamine 2000 (Invitrogen, 11668027) in Opti-MEM (Invitrogen, 31985062). At 48 hours after transfection, coverslips were fixed using 4% paraformaldehyde for 30 minutes and permeabilized with 0.2% Triton in 1× PBS for 10 minutes. Coverslips were stained with SLC13A5 antibody overnight at 4°C (Abnova, H00284111-M06, 1:500) and Alexa Fluor 594 secondary antibody (Thermo Fisher Scientific, A11005, 1:1,000) for 1 hour at room temperature. Coverslips were counterstained with DAPI (500 ng/mL) and mounted with Aqua Poly-mount (18606, Polysciences). Slides were imaged using confocal microscopy (ZEISS LSM 780).

### Citrate analysis.

For citrate uptake analysis, HEK293T cells were maintained and plated in DMEM with 10% FBS, 1% glutamax, and 1% Pen/Strep. At 24 hours after plating, cells were transfected with pAAV9/SLC13A5 or vehicle. After 48 hours, 1 μM of citric acid was added to the cell culture media, and samples of cell culture media were then collected at 30, 60, 90, and 120 minutes after the addition of citrate. Citrate in media was then quantified using GC-MS.

### Blood, CSF, and urine analysis.

For blood citrate analysis, samples were collected from the facial vein using 4 mm lancets (Thermo Fisher Scientific, NC9922361) into tubes containing 0.5 M EDTA to be processed for plasma. Samples were centrifuged at 1,500*g* at 4°C for 12 minutes and then snap-frozen in liquid nitrogen. Blood was drawn from P10 cohort animals 1 mo postinjection. Blood was drawn from mice in the 3 mo cohort prior to treatment and 1 mo postinjection. For terminal CSF harvests, at 6–7 mo postinjection, animals were deeply anesthetized by isoflurane and were secured under a stereotaxic microscope (Nikon, SMZ-800N). A sagittal incision was made in the skin along the back of the skull, carefully separating the subcutaneous tissue and neck muscle through the midline to reveal the cisterna magna. Harvests were completed by puncturing the cisterna magna using a pulled capillary (Thermo Fisher Scientific, NC9544044), collecting between 2 and 7 μL per animal, ensuring no blood contamination within the sample. Samples were immediately snap-frozen in liquid nitrogen. Citrate levels in plasma and CSF citrate were quantified using GC-MS or LC-MS analysis.

Urine was collected via spot collection approximately 2–4 months postinjection. Mice were placed in an empty container lined with sterile parafilm. After approximately 10 minutes, any excreted urine was collected, and samples were snap-frozen in liquid nitrogen. Citrate quantification was completed by the Charles and Jane Pak Center for Mineral Metabolism and Clinical Research at UTSW.

For clinical blood chemistry assessments, at 8 weeks postinjection, blood was collected via facial vein puncture. Samples were allowed to sit at room temperature for 2 hours before being centrifuged at 2,400 × *g* at 4°C for 20 minutes, and then serum was snap-frozen in liquid nitrogen. Samples were submitted to the UTSW Metabolic Phenotyping Core and assessed for blood urea nitrogen (BUN), albumin (ALB), aspartate aminotransferase (AST), and total bilirubin (TBIL).

### Telemetry devices, recordings, and analysis.

Animals were subcutaneously implanted with HD-X02 (Data Systems International) telemetry probes. Dural EEG leads were placed using the coordinates LH: AP +1.0, ML –1.5, RH: AP –2.0, ML +2.0, with EMG being placed in the trapezius muscles in the back of the neck. Following approximately 10 days of recovery from surgery, EEG and EMG recordings were acquired over the course of 60-hour sessions (3 dark cycles, 2 light cycles) conducted 2 times approximately 2 weeks apart. All the recordings took place singly housed in the animal’s home cage with food and water ad libitum. Ponemah (Data Systems International, v6.51) was used to acquire data, and Neuroscore (Data Systems International, v.3.4.0) was used to analyze data. Epileptic spike trains were assessed using a 10 Hz high-pass filter with the automatic spike analysis protocol. A spike train is defined as at least 20 spikes over a minimum of 10-second duration. Activity was automatically determined by implant movement within the cage. These counts were separated into activity during the dark cycles and activity during the light cycles, summed for 2 (light) or 3 (dark) cycles per recording period. Sleep architecture was categorized into active wake, quiet wake, slow wave sleep, or paradoxical sleep based on delta and theta power, muscle tone, and movement using the integrated Rodent Sleep Scoring program. The percentage time spent in each sleep stage was averaged over the 2 recording sessions. Absolute PSD was automatically quantified by the software for 4 frequency bands: delta (0.5–4 Hz), theta (4–8 Hz), alpha (8–12 Hz), and beta (12–30 Hz). Power within the sleep stages was averaged over the 2 recording sessions. Mice that died prior to recording did not have EEG data collected (P10 treatment: *n* = 5 WT+Veh, 4 KO+Veh, 5 KO+LD, 3 KO+HD; 3 mo treatment: *n* = 2 KO+Veh, 1 KO+IT). Additionally, due to automatic light malfunction during a recording session, EEG data were excluded from 3 WT+Veh P10 treated mice.

### Seizure induction.

Seizure susceptibility was assessed using a kindling PTZ induction paradigm. Mice received a 30 mg/kg intraperitoneal injection of PTZ (18682, Cayman Chemicals) dissolved in a sterile 0.9% saline solution every other day for a course of 8 injections (29). Because of telemetry implants, mice were lightly anesthetized using isoflurane for PTZ administration. Animals were monitored for 30 minutes following PTZ injection, and seizure severity was scored using a modified Racine scale (11): Stage 0 (no response); Stage 1 (immobilization); Stage 2 (myoclonic jerks); Stage 3 (clonic seizures); Stage 4 (tonic seizures with rearing); Stage 5 (generalized tonic-clonic seizures); and Stage 6 (seizure-induced death). For seizure latency, time to Stage 3 was recorded. Mice that died on day 1 of injections (P10 treatment: *n* = 1 KO+Veh, 1 KO+LD, 1 KO+HD; 3 mo treatment: *n* = 4 WT+Veh, 1 KO+Veh, 1 KO+ICM) were excluded from PTZ analysis, as this indicates a hypersensitivity to PTZ.

### Necropsy.

At study end, animals were anesthetized via avertin overdose (0.05 mL/g of a 2.5% solution), a terminal blood draw was taken via cardiac puncture, and tissues were collected after perfusion with PBS containing 1 μg/mL heparin. Tissues collected were either snap-frozen in liquid nitrogen for vector distribution or fixed in 10% neutral buffered formalin for histological analysis. Frozen tissues included liver in addition to brain hemisphere subdissected into frontal cortex, brain stem, and cerebellum.

### Vector biodistribution.

Total genomic DNA was purified from frozen tissue samples using a QIACube HT (QIAGEN) and following the 96 DNA QIACube HT Kit protocol. For vector expression, RNA was extracted in QIAzol lysis reagent and chloroform, then purified using a QIACube HT. Extracted RNA samples were treated with DNase I (M0303S, New England Biolabs), and cDNA synthesis was performed using a Transcriptor First Strand cDNA Synthesis Kit (4896866001, Roche). qPCR analysis was performed by Northern Biomolecular Services.

### SLC13A5 immunohistochemistry.

Fixed brains were submitted to the UTSW Histopathology Core for processing, paraffin-embedding, and sectioning. Slides were generated with 5 μm–thick serial sections. For light microscopy, slides were deparaffinized using standard methods, and antigen retrieval was performed with antigen unmasking solution (Vector Laboratories H-3300). The primary antibody used was mouse monoclonal anti-SLC13A5, clone 2G4 (Abnova H00284111-M06, 1:400). The secondary antibody used was biotinylated anti-mouse (Vector Laboratories BA-9200; 1:400). Slides were counterstained with Mayer’s hematoxylin and mounted using Poly-mount Xylene (Polysciences, 24176-120). Slides images were captured using a NanoZoomer and images exported with the ImageScope Software (Leica Biosystems).

For immunofluorescence, following blocking in 5% goat serum, sections were incubated in primary antibodies overnight at 4°C: anti-SLC13A5 (Abnova H00284111-M06, 1:400), anti-S100B (Abcam AB41548, 1:250), and anti-MAP2 (Thermo Fisher Scientific, PA1-10005, 1:1,000). Tissues were then incubated for 1 hour at room temperature in secondary antibodies: Alexa Fluor 488 (Thermo Fisher Scientific, A32731, 1:400), Alexa Fluor 568 (Thermo Fisher Scientific A10037, 1:400), and Alexa Fluor 647 (Thermo Fisher Scientific, A21449, 1:400). DAPI (0.5 ng/μL) was applied for 10 minutes, and slides were coverslipped with aqua polymount. Images were captured at 20× original magnification on a ZEISS AxioScan Z1. To determine the percentage astrocytes and neurons transduced by AAV9/SLC13A5, 2 scorers hand-counted the total neurons (number of MAP2^+^ cell bodies with DAPI nuclei) and the transduced neurons (number of MAP2^+^NaCT^+^ cell bodies with DAPI nuclei) in a defined region of interest (250 μm × 250 μm). Astrocytes were calculated in a similar fashion using S100B^+^ cells. Percentages of cells were averaged between 2 scorers.

### Statistics.

Log-rank (Mantel-Cox) test was used to compare survival curves. Body weight was analyzed using repeated measures 2-way ANOVA, with factors for treatment and days postinjection, and followed with Dunnett’s multiple-comparison test. PTZ was analyzed using repeated measures 2-way ANOVA with factors for injection and genotype or treatment and followed with Dunnett’s multiple-comparison test. Spike train counts, sleep architecture, and citrate were analyzed using a 1-way ANOVA. PSD was analyzed using 2-way ANOVA, with factors for genotype or treatment and waves, and followed by Šídák’s multiple comparison test (genotype) or Dunnett’s multiple-comparison test (treatment). Biodistribution was analyzed using a Student’s unpaired 2-tailed *t* test. For all comparisons, statistical significance was set at *P* ≤ 0.05. Data were analyzed and graphed using GraphPad Prism software (v. 10.4.2).

### Study approval.

All animal experiments were reviewed and approved by the UTSW Institutional Animal Care and Use Committee.

### Data availability.

All data associated with this study are available in the main text or the supplement, and additional study details are available from the authors upon request. We previously reported analysis of sleep architecture and power spectra from WT+Veh and KO+Veh mice in the P10 cohort in Adams et al. (2). In the current manuscript, new analysis of KO+Veh, KO+LD, and KO+HD mice from the P10 cohort is reported. Any plasmids, vectors, or animal models used in this study are available upon request through a material transfer agreement. Values for all data points in graphs are reported in the Supporting Data Values file.

## Author contributions

RMB conceptualized the study. LEB, RMA, MKS, ITG, KK, SKH, and MME performed experiments. LEB, RMA, and RMB analyzed data. ML helped with statistical analysis. RMB supervised all activities of the study. LEB, RMA, and RMB wrote the original manuscript draft. Co-first author order was determined by who conducted the majority of the data analysis. All authors reviewed the manuscript.

## Conflict of interest

RMB is an inventor on the SLC13A5 vector design (US 2023.0285595) and has received income related to that invention.

## Funding support

- TESS Research Foundation to RMB.
- Taysha Gene Therapies to RMB.

## Supplementary Material

Supplemental data

Supporting data values

## Version 1 02/19/2026

In-Press Preview

## Version 2 04/15/2026

Electronic publication
