# Supplementary material for: AAV-mediated gene therapy in a model of SLC13A5 citrate transporter disorder rescues epileptic and metabolic phenotypes
Source: J Clin Invest. 2026 Feb 19;136(8):e197503. doi: 10.1172/JCI197503 (PMC13078891; doi:10.1172/JCI197503)
Supplement: Supplemental data [file jci-136-197503-s303.pdf]

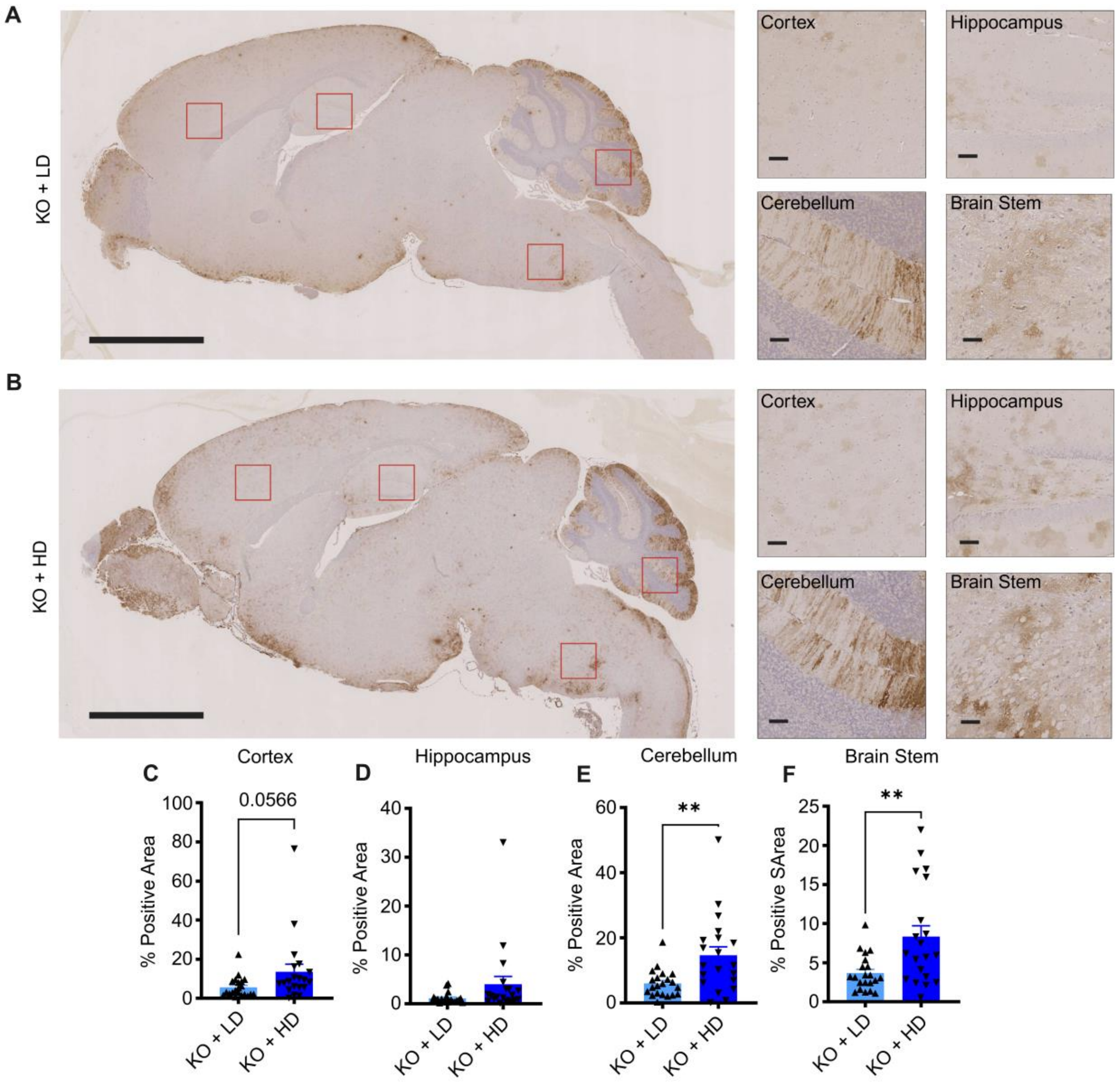

Supplemental Figure 1: Regional brain distribution of vector derived NaCT expression of P10 injected mice. (**A-B**) Representative images of mouse brain stained with SLC13A5/NaCT antibody of a low dose (**A**) and high dose (**B**) treated mouse collected 4 mo post-treatment. Regional images represent the cortex (top left), hippocampus (top right), cerebellum (bottom left), and brain stem (bottom right). *Scale bar 3mm for overview images, 100 µm for regional image*s. (**C-F**) Percent positive NaCT staining in the cortex (C), hippocampus (**D**), cerebellum (**E**), and brain stem (**F**). Student's t-test, $^{**}p<0.01$. n=21 KO+LD, 20 KO+HD. Data shown as Mean ± SEM.

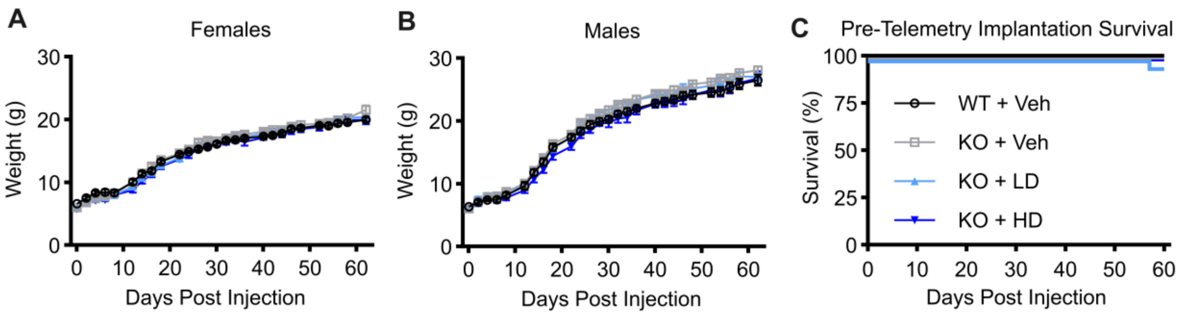

Supplemental Figure 2: P10 IT treatment of AAV9/SLC13A5 resulted in normal weight gain and survival in KO mice. WT and KO Slc13a5 littermates were treated with vehicle, 2e11 vg (LD) or 8e11 vg (HD) at post-natal day 10. (**A-B**) Weight gain in females (**A**) and males (**B**) following treatment up to 60 days post injection. (**C**) Survival following treatment until telemetry implant surgery, 60 days post injection. n = 12F/13M WT+Veh, 14F/12M KO+Veh, 13F/12M KO+LD, 11F/11M KO+HD. Data shown as Mean ± SEM.

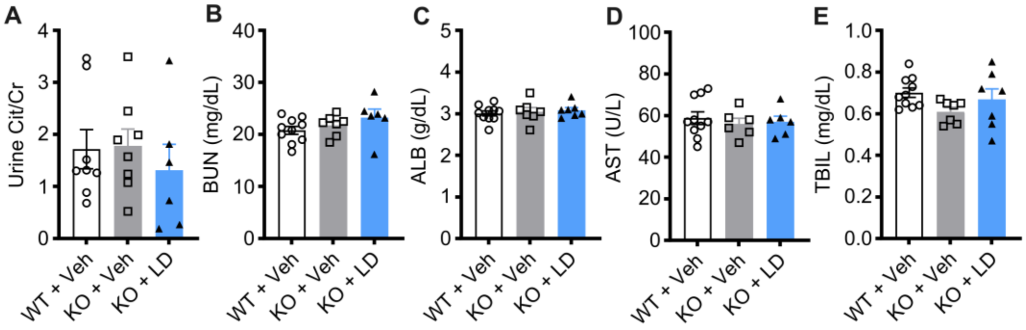

Supplemental Figure 3: Urinary citrate levels with preserved kidney and liver function after AAV9/SLC13A5 administration. WT and KO Slc13a5 littermates were treated with vehicle or 2e11 vg (LD) at post-natal day 10. (A) Urinary citrate concentration, normalized to urinary creatinine collected 2-4 mo post-treatment. (B-D) Clinical blood chemistry in serum assessing blood urea nitrogen (BUN; B), albumin (ALB; C), aspartate aminotransferase (AST; D), and total bilirubin (TBIL; E) collected 2 mo post-treatment. n=8-10 WT+Veh, 8=KO+Veh, 6-7 KO+LD. Data shown as Mean ± SEM

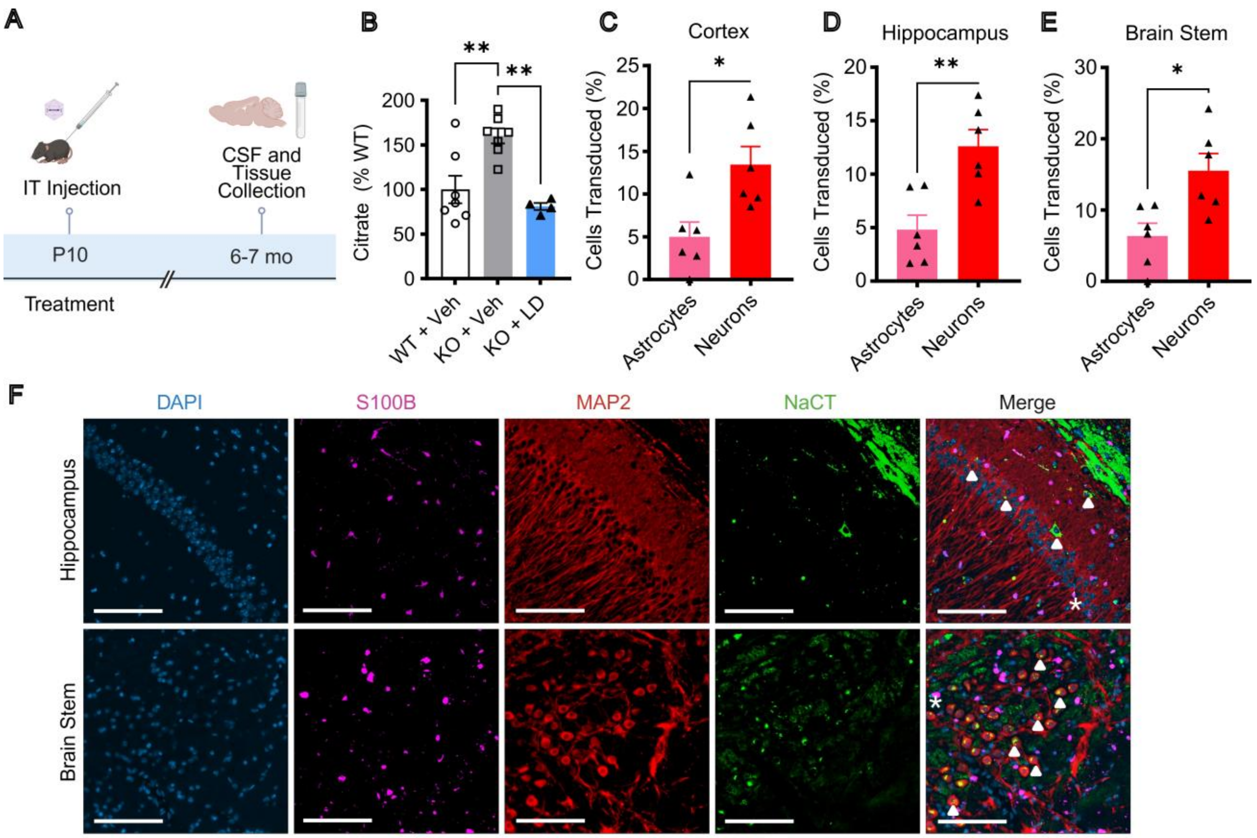

Supplemental Figure 4: P10 IT administration of AAV9/SLC13A5 decreased CSF citrate in KO mice and resulted in higher neuron versus astrocyte transduction. (**A**) Schematic of the efficacy study in *Slc13a5* KO mice IT injected at P10 with vehicle, or 2e11 vg (LD). (**B)**. LC-MS analysis of citrate in CSF at 6-7 mo post-injection, relative to vehicle treated WT mice. One-way ANOVA as compared to KO+Veh with Dunnett's Multiple Comparison's test, $^{**}p<0.01$. n=7 WT+Veh, 7 KO+Veh, 4 KO+LD. (**C-E**) Brains collected 6-7 mo post-treatment were co-stained for transduced cells (NaCT), astrocytes (S100B), neurons (MAP2) and nuclei (DAPI). Percent S100B+ cells and MAP2+ cells double positive for NaCT in the cortex (**C**), hippocampus (**D**), and brain stem (**E**) in KO+LD. Student's t-test, $^{*}p<0.05$, $^{**}p<0.01$, n=6. (**F**) Representative images of the hippocampus (top) and brain stem (bottom). Arrowhead indicates MAP2+NaCT+ cell; asterisk indicates S100B+NaCT+ cell. Data shown as Mean ± SEM. *Scale bar 100 µm.*

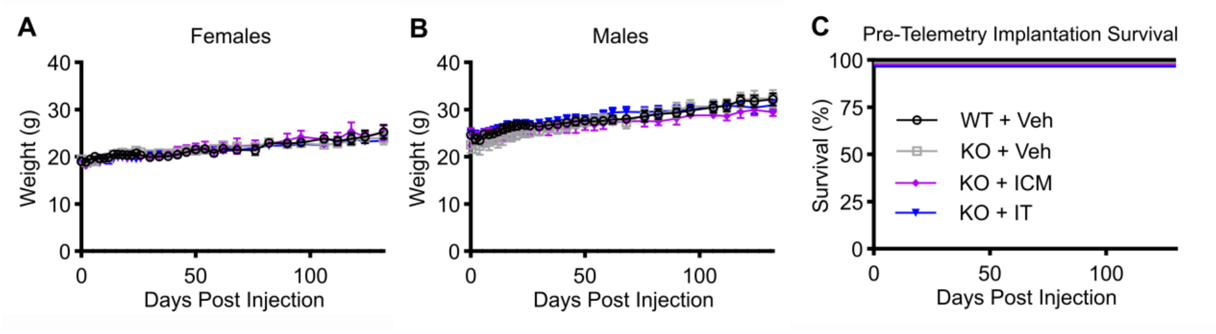

Supplemental Figure 5: ICM and IT delivery of AAV9/SLC13A5 at 3 mo resulted in normal weight gain and survival in KO mice. WT and KO Slc13a5 littermates were treated with vehicle or 8e11 vg via ICM or IT injection at 3 mo of age. (**A-B**) Weight gain in females (**A**) and males (**B**) following treatment up to 130 days post injection. (**C**) Survival following treatment until telemetry implant surgery, 130 days post injection. n = 5F/6M WT+Veh, 6F/6M KO+Veh, 3F/4M KO+ICM, 4F/3M KO+IT. Data shown as Mean ± SEM.

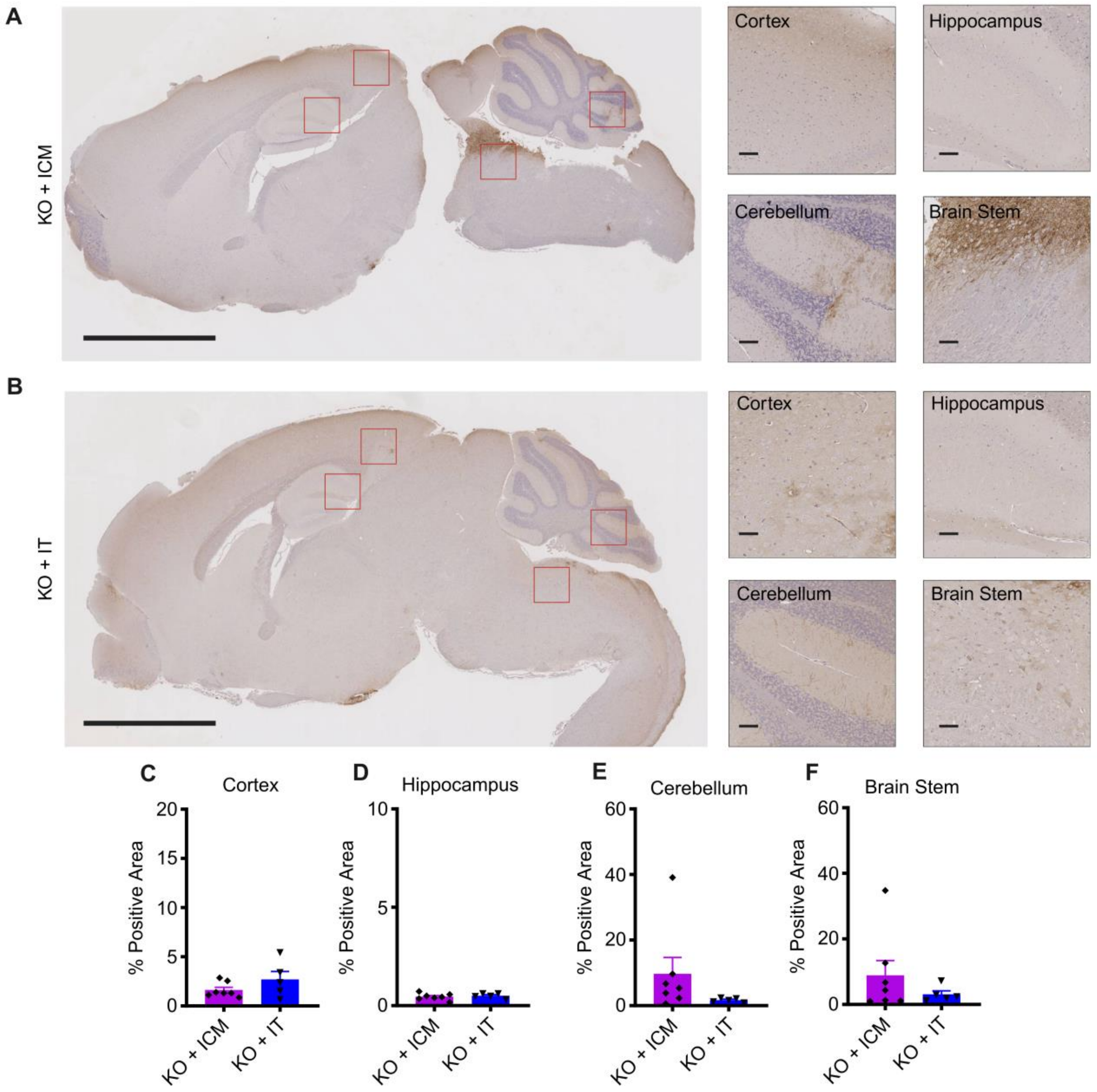

Supplemental Figure 6: Regional brain distribution of vector derived NaCT expression in 3 mo injected mice. (A-B) Representative images of mouse brain stained with SLC13A5/NaCT antibody of an ICM (**A**) and IT (**B**) treated mouse collected 6 mo post-treatment. Regional images represent the cortex (top left), hippocampus (top right), cerebellum (bottom left), and brain stem (bottom right). *Scale bar 3mm for overview images, 100 µm for regional images.* (**C-F)** Percent positive NaCT staining in the cortex (**C**), hippocampus (**D**), cerebellum (**E**), and brain stem (**F**). N=7 KO+ICM, 5 KO+IT. Mean ± SEM.

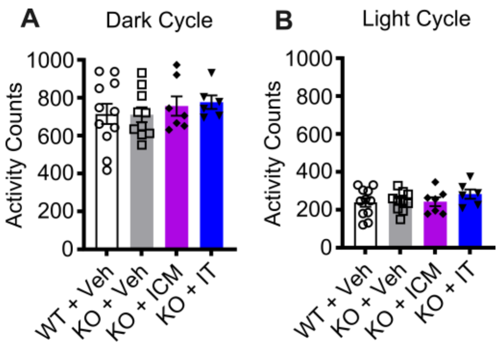

Supplemental Figure 7: Adult treated *Slc13a5* KO mice had normal activity levels at 8 mo of age (**A-B**) Overall activity counts during the dark (**A**) and light (**B**) cycles in WT+Veh, KO+Veh, KO+ICM, and KO+IT treated mice. n = 11 WT+Veh, 10 KO+Veh, 7 KO+ICM, 6 KO+IT. Data shown as Mean ± SEM.

Supplemental Figure 8

UNIVERSITY OF NORTH CAROLINA VECTOR CORE

Quality Control Summary

Lot #	LAV55-conc

Test by qPCR

Test #	Titer, vg/mL	Analyst	Date	File
1	1.55E+14	PZ	04/04/2018	20180404-1428-ghbh-pz

PAGE analysis

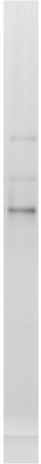

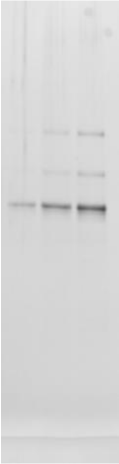

Loaded 5.00E+09 vg 4980E std 2e9vg 5e9vg 1e10vg
Calculated 4.30E+09 vg

Analyst	Ping Zhang
Date	04/02/2018
Reference #	20180402-silver

SEM

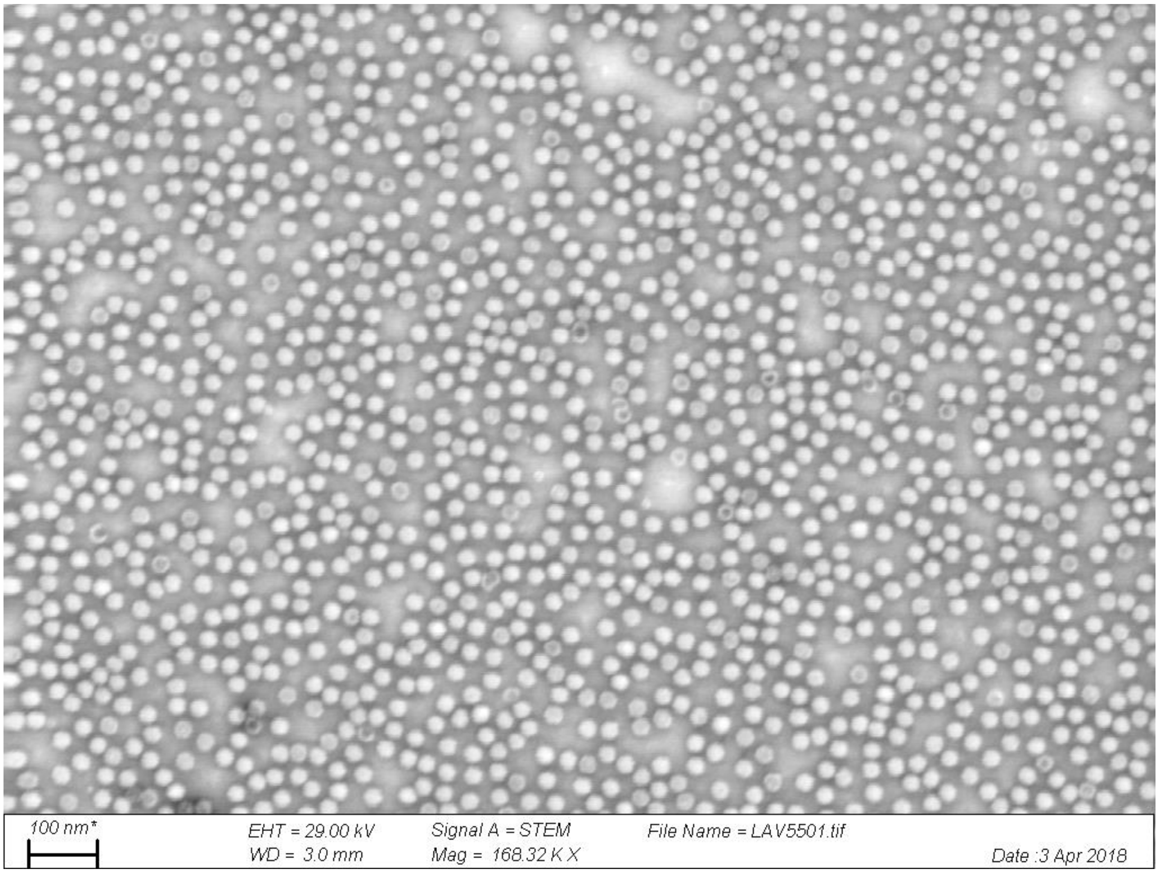

90% full

Analyst	Ping Zhang
Date	04/03/2018
Reference #	20180403-LAV55-01

Quality Control Summary

Lot #	LAV131	Name	SLC13A5

Test by qPCR

Test #	Titer, vg/mL	Analyst	Date	File
1	1.38E+14	PZ	07/08/2020	20200708-1351-ghbh-pz

PAGE analysis

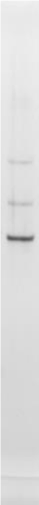

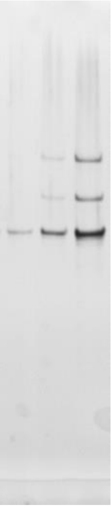

Loaded 5.00E+09 vg 4980E std 2e9vg 5e9vg 1e10vg
Calculated 5.10E+09 vg

Analyst	Ping Zhang
Date	06/29/2020
Reference #	20200629-silver

SEM

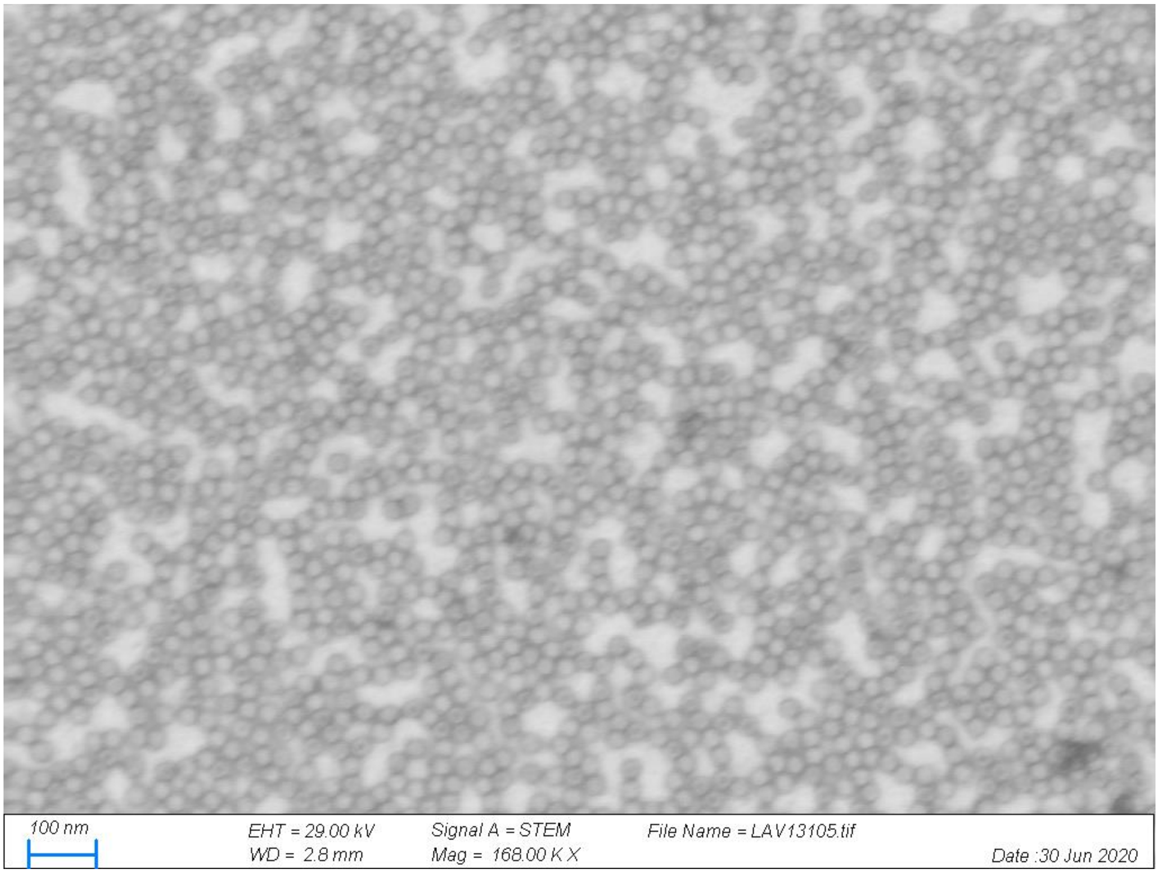

92% full

Analyst	Ping Zhang
Date	06/30/2020
Reference #	20200630-LAV131-05

Supplemental Table 1: Study animal numbers

Age of Injection	Cohort	Group	EEG Recording	PTZ Induction	Bio-distribution	Urine Analysis	CBC Analysis	CSF Analysis
P10	1	WT + Veh	17 (9F/8M)	20 (10F/10M)	-	-	-	-
		KO + Veh	22 (11F/11M)	21 (10F/11M)	-	-	-	-
		KO + LD	20 (10F/10M)	19 (10F/9M)	15 (8F/7M)	-	-	-
		KO + HD	19 (9F/10M)	18 (9F/10M)	14 (7F/7M)	-	-	-
	2	WT + Veh	-	-	-	8 (6F/2M)	10 (6F/4M)	-
		KO + Veh	-	-	-	8 (4F/4M)	7 (4F/3M)	-
		KO + LD	-	-	-	7 (4F/3M)	7 (4F/3M)	-
	3	WT + Veh	-	-	-	-	-	7 (4F/3M)
		KO + Veh	-	-	-	-	-	7 (4F/3M)
		KO + LD	-	-	-	-	-	5 (2F/3M)
3 mo	4	WT + Veh	11 (5F/6M)	7 (5F/2M)	-	-	-	-
		KO + Veh	10 (4F/6M)	9 (4F/6M)	-	-	-	-
		KO + ICM	7 (3F/4M)	6 (3F/3M)	5 (3F/2M)	-	-	-
		KO + IT	6 (4F/2M)	6 (4F/2M)	4 (3F/1M)	-	-	-

CBC = clinical blood chemistry
